# Structural and functional studies of the metalloregulator Fur identify a promoter-binding mechanism and its role in *Francisella tularensis* virulence

**DOI:** 10.1038/s42003-018-0095-6

**Published:** 2018-07-17

**Authors:** J. Pérard, S. Nader, M. Levert, L. Arnaud, P. Carpentier, C. Siebert, F. Blanquet, C. Cavazza, P. Renesto, D. Schneider, M. Maurin, J. Coves, S. Crouzy, I. Michaud-Soret

**Affiliations:** 1 Univ. Grenoble Alpes, CNRS, CEA, BIG-LCBM, 38000 Grenoble, France; 2 Univ. Grenoble Alpes, CNRS, CHU Grenoble Alpes, Grenoble INP, TIMC-IMAG, 38000 Grenoble, France; 3 Univ. Grenoble Alpes, CNRS, CEA, IBS, 38000 Grenoble, France

## Abstract

*Francisella tularensis* is a Gram-negative bacterium causing tularaemia. Classified as possible bioterrorism agent, it may be transmitted to humans via animal infection or inhalation leading to severe pneumonia. Its virulence is related to iron homeostasis involving siderophore biosynthesis directly controlled at the transcription level by the ferric uptake regulator Fur, as presented here together with the first crystal structure of the tetrameric *F. tularensis* Fur in the presence of its physiological cofactor, Fe^2+^. Through structural, biophysical, biochemical and modelling studies, we show that promoter sequences of *F. tularensis* containing Fur boxes enable this tetrameric protein to bind them by splitting it into two dimers. Furthermore, the critical role of *F. tularensis* Fur in virulence and pathogenesis is demonstrated with a *fur*-deleted mutant showing an attenuated virulence in macrophage-like cells and mice. Together, our study suggests that Fur is an attractive target of new antibiotics that attenuate the virulence of *F. tularensis*.

## Abstract (web summary)

Pérard et al. report the structure of *Francisella tularensis* Fur (FtFur) with its physiological cofactor Fe^2+^, and show that FtFur is important for virulence. This study identifies a promoter-driven tetramer splitting mechanism that may provide insight into future antibiotics development.

## Introduction

**Fig. 1:**
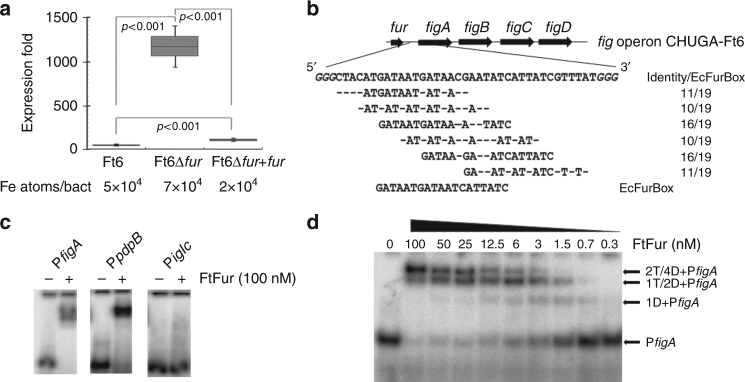
FtFur regulates *fig* operon by recognition of DNA FurBox. **a** qRT-PCR showing the absence of *fur* transcripts in the CHUGA-Ft6∆*fur* strain, with a 16S RNA standard as a control, Fur (Ft6 and Ft6∆*fur* + *fur*) repressed the transcription of *figA*. This repression is abolished in the absence of Fur (Ft6∆*fur*). The data correspond to two independent experiments made in triplicate. *P* values were calculated using the Student's *t* test. Iron concentration was measured by ICP-AES (error under 1%) from 2 mL bacterial culture (see Methods section) and number of Fe atoms per bacteria has been deduced. **b** Organization of the *fig* operon and sequence of the *fur-figA* intergenic region (P*fig*A). The identical bases between P*fig*A and EcFurBox are indicated underneath showing overlapping FtFur binding sites. **c** Evaluation of the ability of FtFur to bind identified or predicted Fur boxes and estimation of the apparent Kds (for DNA seq of each promoter see Supplementary Fig. 1). **d** EMSA of FtFur in the presence of the 43 bp P*fig*A sequence. The proposed stoichiometry is written on the figure: D corresponding to dimer and T to tetramer

F*rancisella tularensis* is a small, highly infectious Gram-negative bacterium, causing the zoonotic disease tularaemia^1^. This species is currently divided into three subspecies, including subsp. *tularensis* (type A strains), subsp. *holartica* (type B strains) and subsp. *mediasiatica*^2^. Only type A and type B strains of *F. tularensis* are known to cause tularaemia in humans. A large number of animal species can be infected with this pathogen, but lagomorphs and small rodents are considered the primary sources of human infections. The disease may also be transmitted through arthropod bites, mainly *Ixodidae* ticks and mosquitoes. *Francisella tularensis* also survives for prolonged periods in the environment, and humans can be infected through contact with contaminated soil or water. Because a few bacteria inhaled through aerosols may induce an acute severe pneumonia, with a mortality rate of 30% or more, *F. tularensis* has been classified as a potential category A biothreat agent by the Center for Disease Control and Prevention^3^. No effective vaccine is currently licenced for human or animal use, and a few antibiotic compounds are used as first-line drugs in tularaemia patients. Alternative treatments are urgently needed both to improve the prognosis of patients with severe diseases, and also to improve our preparedness to the intentional release of resistant strains of this pathogen in the context of bioterrorism^4,5^. Although numerous genes have been shown to be important for the pathogenesis and virulence of *F. tularensis*, there is still a blatant lack of knowledge about central biological functions such as iron homeostasis^6,7^ and metalloregulators^8,9^. As a facultative intracellular bacterial pathogen, *F. tularensis* multiplication and virulence depend on the host cell iron pool^10^. Indeed, a major defence strategy used by infected eukaryotic organisms is to withhold this metal by sequestering free iron. In reaction to iron starvation, *F. tularensis* is able to secrete an iron chelator structurally similar to the polycarboxylate siderophore rhizoferrin^11,12^. The *figA* gene (also called *fslA*), involved in the siderophore synthesis, but also the *figE* gene (*fslE)*, responsible for its uptake, have been characterized to play an important role in the virulence and/or intracellular replication of this pathogen^9,12,13^. These genes belong to the locus *figABCDEF* (*fig* for *Francisella* iron-regulated genes)^14,15^ (Fig. 1). The *fig* operon is regulated by the ferric uptake regulator Fur, which is supposed to bind to the *fur-figA* intergenic region that contains a specific sequence called a FurBox (Fig. 1a), although such direct interaction has not yet been demonstrated. The Fur protein is a global transcriptional regulator that senses iron status and controls the expression of genes involved in iron homeostasis, virulence and oxidative stresses^16–18^.

In the present study, to go further in the in vitro and in vivo characterization of the properties of the *F. tularensis* Fur (FtFur) protein, we used a virulent *F. tularensis* subsp. *holarctica* strain, a Type B biovar I, referred to as CHUGA-Ft6. This strain was isolated from a blood sample from a French patient suffering from a typhoidal form of tularaemia^19^. Interestingly, comparing FtFur to Fur from *Escherichia coli* (EcFur), *Pseudomonas aeruginosa* (PaFur), *Legionella pneumophila* (the agent of legionellosis, LpFur) and *Yersinia pestis* (the agent of plague, YpFur), we have evidenced that these proteins can be discriminated by their quaternary structure in solution^20^. EcFur and YpFur belong to the group of the commonly accepted dimers, while FtFur, PaFur and LpFur belong to a group of tetramers. A structural zinc in a cysteine-rich site (site 1) has been characterized in many Fur proteins including FtFur^16,21,22^. In addition, the Fur proteins need metallic dications such as Co^2+^, Mn^2+^ or Fe^2+^ in a regulatory site (site 2) to be activated for the binding to DNA^20^.

Here, we present, to our knowledge, the first crystal structure of a tetrameric Fur protein in the presence of its physiological cofactor, the ferrous ion. This structure sheds light on the metal-binding sites and corresponds to two intertwined pre-activated dimers. We demonstrate the direct interaction of the protein with the promoter region controlling expression of the genes involved in siderophore synthesis and identify essential residues in this interaction. In addition, owing to the coupling of computer models and free energy calculations with cross-link experimental studies, we bring evidence for a DNA-driven tetramer splitting mechanism mediated by specific promoter sequences, and leading to the formation of two Fur dimer–DNA complexes. Finally, the critical role of FtFur in bacterial virulence and pathogenesis is demonstrated using a *fur*-deleted CHUGA-Ft6 mutant (Ft6Δ*fur*), which shows an attenuated virulence, both in murine macrophage-like cells and in mice, reinforcing that FtFur can be thus defined as a crucial anti-virulence target.

## Results

### Fur is directly involved in *F. tularensis* iron homeostasis

A ∆*fur* mutant was already generated in the virulent Schu S4 strain (subsp. *tularensis*) to demonstrate that siderophore production is regulated by FtFur in *F. tularensis*^9^. However, the direct involvement of FtFur in virulence has never been reported to our knowledge. We have constructed the CHUGA-Ft6∆*fur* strain by the allelic exchange method and deletion was confirmed by quantitative real-time polymerase chain reaction (qRT-PCR) and sequencing as we did not detect any *fur* transcript in Ft6∆*fur* (Supplementary Fig. 1). Using this approach, we demonstrated that the siderophore synthesis is under the direct control of FtFur in CHUGA-Ft6 strain. CHUGA-Ft6∆*fur* shows an approximately 25-fold higher level of *figA* transcript when cultured in iron-replete conditions compared to the wild-type (WT) strain. The WT phenotype, that is Fur transcriptional repression of the *fig* operon genes, is recovered when the WT *fur* is expressed in trans to complement the *fur* deletion (CHUGA-Ft6∆*fur* + *fur*) (Fig. 1a). This means that siderophore production is repressed by FtFur in the presence of iron and derepressed in the absence of the protein. Inductively coupled plasma atomic emission spectroscopy (ICP-AES) quantification of the bacterial iron concentration showed that, under our culture conditions, the CHUGA-Ft6∆*fur* strain accumulates 1.6-fold more iron than the WT (Fig. 1a). These data strongly suggest that FtFur can bind the *fur-figA* intergenic region that contains sequences closely related to the EcFurBox identified in *E. coli* (Fig. 1b). Only a few Fur boxes were identified in *Francisella* genome, compared to *E. coli*, in the promoter of *figA*, *pdpB* (coding for the pathogenicity determinant protein PdpB) and *iglC* (coding for the pathogenicity island protein IglC) both in Schu S4^14^ and CHUGA-Ft6. Then, electrophoretic mobility shift assay (EMSA) with manganese-activated FtFur have been performed on consensus EcFurBox and on P*figA*, P*pbp*B and P*igl*C sequences (Fig. 1c and Supplementary Fig. 2).

FtFur binds with a very high affinity to EcFurBox when activated with Co(II) (Kdapp = 9 nM^20^) and to the P*figA* promoter (estimated Kdapp = 5 nM) and with a low affinity to P*pbpB* (averaged estimated Kdapp = 100 nM; Fig. 1c and Supplementary Fig. 2C). In contrast, and while *iglC* gene expression is also found up-regulated under iron-restricted conditions in *F. tularensis*^14^, no binding is detected indicating the absence of direct regulation by Fur. The migration on EMSA gel of the FtFur/P*fig*A complex shows a composite pattern with three successive bands assigned to the binding of one to two tetramers (or one to four dimers) (Fig. 1d and Supplementary Fig. 2B) which appear as the protein concentration is increased. This suggests that FtFur could bind to several predicted Fur boxes in the sequence (Fig. 1b). Indeed, using a shorter version (P*figA*S) of P*figA*L, the main species detected corresponds to one dimer bound to DNA (Supplementary Fig. 2B).

Western blot experiments suggest that the estimated 3000 protein subunits/bacteria may be mainly present as a tetramer in vivo (see Supplementary Fig. 2F, G) and that hydrogen peroxide (H_2_O_2_) treatment (1 mM for 4 h) does not impact this amount. This is not surprising considering the high stability of the tetramer in solution. This copy number is in the same range as described in *E. coli* or *Vibrio cholerae* (5000 and 2500 subunits/bacteria estimated, respectively, in normal growth conditions)^23,24^. Considering the number of 50,000 total iron atoms/bacteria quantified by ICP-AES (Fig. 1a) and the volume of CHUGA-Ft6 around 10^−15^ L, 5 µM of FtFur subunit and 80 µM total iron are expected. Assuming micromolar range Kd for (Fe-FtFur) as found in the literature for *E. coli* (1–10 µM for Fe-EcFur^25^), we can expect that a pool of metallated Fur tetramer exists in the cell prior to association with the few DNA target present in *F. tularensis*.

### Fe-FtFur and Mn-FtFur contain intertwined pre-activated dimers

**Fig. 2:**
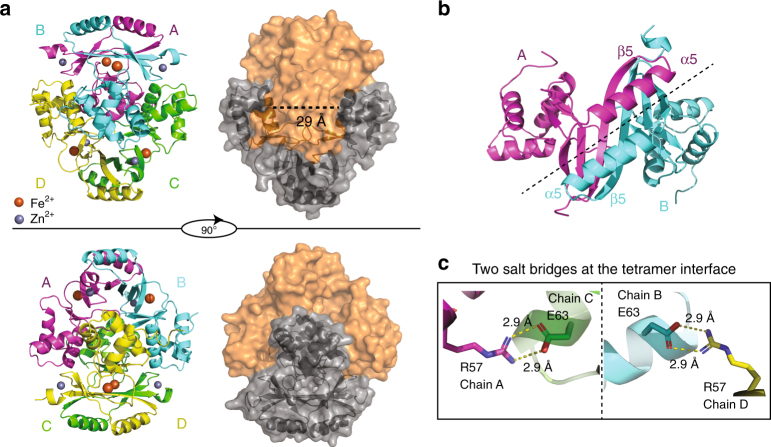
Structure of FtFur at 1.8 Å resolution in the presence of physiological iron Fe^2+^. **a** Fe-FtFur structure solved by SAD at 1.8 Å under anaerobic conditions in the presence of Fe^2+^. The cartoon model presents the four chains, labelled A–B–C–D. Surface representation indicates the dimer/dimer interface and the distance between two recognition helices (29 Å). **b** Symmetry at the dimer/dimer interface between two monomers involving helix α5 and strand β5. **c** One of the most important interactions suggested by the structure is a salt bridge between Arg57 and Glu63

Recombinant FtFur was purified as a tetramer containing one equivalent of Zn(II) per subunit^20^. FtFur was crystallized in the presence of Mn(II) as MnCl_2_ or Fe(II) as (NH_4_)_2_Fe(SO_4_)_2_, the latter under anaerobic conditions. The structure of Mn-bound FtFur was obtained from purified protein metalled with Mn at high concentration before crystallization in the presence of Mn(II) and was determined ab initio at 1.7 Å resolution by the single-wavelength anomalous diffraction (SAD) method. This structure was used to determine that of Fe(II)-bound FtFur at 1.8 Å resolution by molecular replacement (see X-ray data in Supplementary Fig. 3 and Supplementary Tables 1 and 2). Both Mn(II)-bound and Fe(II)-bound proteins have similar overall structures appearing as a compact tetramer made of a dimer of dimers per asymmetric unit (α-carbon root mean square deviation (r.m.s.d.) of 0.271 Å between the two structures). The main differences come from disordered N-terminal and C-terminal residues. Thus, the structure of Fur containing the physiological activator metal, namely Fe(II)-bound FtFur, the first one described to date, will be used for a detailed description (Fig. 2a). Among the total 140 residues of the protein, 131 to 133 were resolved per chain. Each subunit presents secondary structure elements similar to those found in other Fur structures. It consists of a N-terminal DNA-binding domain (residues 7–82) composed of a winged helix–turn–helix motif in which α4 is the DNA recognition helix. A short hinge connects the DNA-binding domain to the C-terminal dimerization domain (residues 89–138). The dimerization domain consists of three antiparallel β-strands (β3 to β4) and two α-helices (α5 to α6), α5 intersecting between β4 and β5 (Fig. 2a and Supplementary Fig. 4). The dimeric interface is mediated by β5 from each subunit forming an antiparallel β-sheet, part of a six-stranded β-sheet in the dimer (Fig. 2b).

The two dimers in the tetramer structure are nearly identical (α-carbons r.m.s.d. = 0.318 Å between the two dimers) with an almost perfect superposition of the secondary structure elements. The interaction between the two dimers through their DNA-binding domains is stabilized by H-bonds involving atoms of the DNA recognition helices (α4) of chains AB and CD (for chains A and C: Gln61_C_ Hε/Ser64_A_ O; Ser64_C_ Hγ/Ser64_A_ Oγ; Ser64_C_ O/Gln61_A_ Hε and Arg57_C_ Hh/Glu63_A_ Oε salt bridge, and equivalently for chains B and D). The two salt bridges between Arg57 and Glu63 constitute the most important interactions (Fig. 2c). These interactions combined with an interface area of 2830 Å^2^ between the dimers AD and BC (PISA^26^) explain the high stability of the tetramer in solution (Fig. 2d). For comparison, in a previous work, we demonstrated that PaFur, initially described as a crystallographic dimer^27^, was tetrameric in solution^20^ with a substantially lower predicted interface area of 2120 Å^2^.

Each dimer is in a closed conformation with the wing in 'inside' positions corresponding to the 'active' form in which the DNA-binding domains are prepared to bind target DNA^20^. However, the dimer–dimer interactions naturally prevent any kind of interaction with DNA through the recognition helices. Indeed, Tyr56 and Arg57 (the one involved in the salt bridge stabilizing the tetramer), both present in the recognition helix, are highly conserved residues known to have base-specific interaction with DNA^28–30^. We thus hypothesize that the metalled FtFur tetramer structure is a pre-activated form of the protein. The mechanism of pre-activated tetramer disruption driven by DNA is conceptually of interest.

### First structural description of an iron substituted Fur

**Fig. 3:**
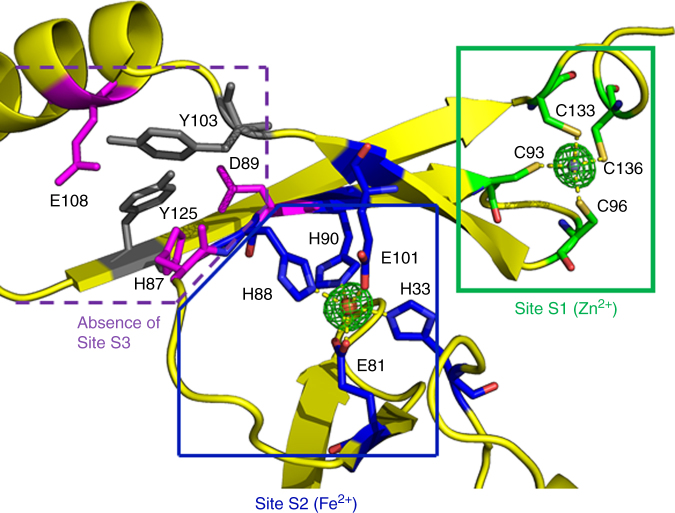
Metal binding site in Fe-FtFur. Focus on one monomer extract on Fe-FtFur showing S1 and S2 sites simultaneously together with the unfilled S3 site

X-ray fluorescence spectra indicated that Fur crystals contain two metal species: one is the expected Zn and the second is the metal added during crystallization, that is, Mn(II) or Fe(II) (Supplementary Fig. 3A). The structures of Mn-FtFur and Fe-FtFur are similar and confirm the presence of one Zn^2+^ and one Mn^2+^ or Fe^2+^ per subunit. Zn^2+^ in structural site S1 is coordinated by four sulphur atoms from two pairs of cysteines in CX_2_C motifs (Cys93-Cys96 and Cys133-Cys136) (Table 1). It connects the short C-terminal helix α5 to the β-sheet of the dimerization domain (Supplementary Fig. 4). The presence of S1 in Fe-FtFur but not in PaFur demonstrates that zinc is not a prerequisite for tetramer formation. The second site S2 binds either Mn^2+^ or Fe^2+^. The metal ion adopts a distorted octahedral geometry with a 3N–3O coordination sphere (Fig. 3). S2 connects the DNA-binding domain (His33 and Glu81 (bidentate) and the dimerization domain which provides three ligands (His88, His90 and Glu101). It is described as the essential 'regulatory' site, present in all known activated Fur structures. H33A-H90A double mutations in FtFur S2 provoke a total inactivation of the protein in vitro (Supplementary Fig. 5C). The Fur structures containing an S2 site filled with Zn^2+^ show some variation in the coordination sphere. This flexibility may be explained by the preference of Zn^2+^ for a tetrahedral geometry compared to Fe^2+^, found physiologically in S2, which favours a hexacoordinated octahedral environment with N/O ligands. Ab initio quantum chemical geometry optimizations of models of the S2 site using DFT with B3LYP hybrid functional and 6–31 G(d) basis set have been performed with bound Mn^2+^ or Fe^2+^. The similar optimized geometries, with a larger coordination sphere for Mn^2+^ than for Fe^2+^, validate the X-ray structures (Fig. 3, Supplementary Fig. 4A–D and Supplementary Table 3).

**Table 1:** Metal coordination from X-ray and DFT calculations

**Atoms**	**Distance**	**Å**
**Site S1**	**Zn**	**Zn**
Cys93/96/133/136S	2.3	2.3
**Site S2**	**Fe**	**Mn**
	**X-ray / DFT**	**X-ray / DFT**
His33 NƐ2	2.3 / 2.20	2.3 / 2.26
Glu81 OƐ1	2.2 / 2.25	2.3 / 2.43
Glu81 OƐ2	2.3 / 2.36	2.3 / 2.32
His88 NƐ2	2.3 / 2.22	2.3 / 2.25
His90 NƐ2	2.2 / 2.18	2.3 / 2.22
Glu101 OƐ1	2.0 / 2.05	2.3 / 2.10

Bond distances for sites S1 and S2 ligands deduced from the X-ray structures and calculated by DFT

Some structures of Fur or Fur-like proteins (such as HpFur and PaFur) revealed the presence of a third metal-binding site (S3) involving four conserved residues 2 His, 1 Asp and 1 Glu^22,27^. In FtFur, Tyr125 is found in place of one very conserved His. The structure shows that the phenol group makes H-bonds with the other putative ligands preventing metal binding in the position where a metal ion was expected. Accordingly, the structures of FtFur do not display any S3 site (Fig. 3 and Supplementary Fig. 4).

In summary, the crystal structures of FtFur highlighted the presence of two metal ions per subunit: one structural Zn^2+^, already present in the non-activated protein as purified, and either one Mn^2+^ or one Fe^2+^, its physiological activator, with identical ligands in the regulatory site of similar geometry. Metalled FtFur behaves as a dimer of pre-activated dimers with the DNA-binding domains forming a kind of crown with interacting recognition helices through two salt bridges between Arg57 and Glu63 together with other weaker interactions.

### Quaternary structure of FtFur in the presence of the FurBox

**Fig. 4:**
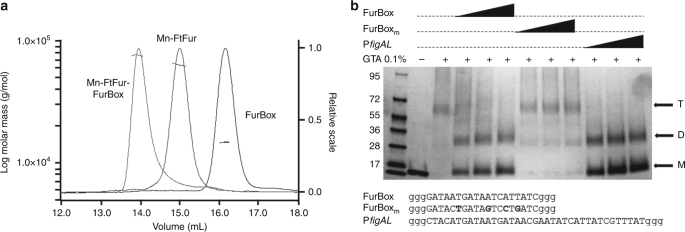
Quaternary structure of Mn-FtFur in the presence of FurBox analysed by SEC-MALLS-RI and cross-link assay. **a** SEC-MALLS-RI data of Mn-FtFur and the FurBox analysed alone or as a complex in the presence of 1 mM MnCl_2_. Data are normalized by RI scale with sample concentrations ranging between 4 and 6 mg mL^−1^. FtFur/FurBox gives a MW of 78 ± 2 kDa, Mn-FtFur gives a MW of 64 ± 1 kDa and FurBox gives a MW of 15 ± 0.5 kDa. **b** Cross-link assay with GTA on SDS-PAGE 4–20% acrylamide in TGS buffer. A dimer (D) was trapped in the presence of specific Fur boxes (FurBox and P*figAL*), while FtFur exists as a tetramer (T) in the absence of DNA or in the presence of mutated FurBox_m_

Size-exclusion chromatography coupled to multi-angle laser light scattering with online refractometer (SEC-MALLS-RI) was used to investigate the behaviour of FtFur in the presence of DNA. As shown in Fig. 4a, in the presence of FurBox the protein eluted at a lower volume than the protein alone or the FurBox alone. The deduced molecular weight of the corresponding peak is 74 ± 2 kDa, fitting with a complex between tetrameric FtFur (64 kDa) and the FurBox duplex (15 kDa). This can be interpreted as the binding of FtFur to DNA as a tetramer or as two dimers.

The evolution of the purified tetrameric FtFur in the presence of DNA was then analysed by cross-link experiments using 0.1% glutaraldehyde (GTA). Under denaturing conditions, in the absence of DNA (Fig. 4b), the main detected band corresponds to a species with a molecular weight of approximately 62 kDa, in very good agreement with the size of a covalently bound FtFur tetramer. After cross-link in the presence of the EcFurBox, only two bands were detected corresponding to the monomer and to a dimeric form of the protein, respectively. Mutations of the FurBox (FurBox_m_, see Supplementary Fig. 2) targeting four bases previously shown to be crucial for the specific Fur/DNA interactions^31^, three of them being involved in interactions with Tyr56^28,29^, resulted in the conservation of the tetramer without apparition of dimers.

The monomer (M)/dimer pattern was also obtained with P*figA*. These results demonstrate that tetrameric FtFur splits into dimers in the presence of specific DNA contrary to the dissociation of PaFur previously observed with non-specific DNA^20^. Besides, they strongly suggest that FtFur binds the FurBox as dimers in vitro.

### MALLS and SAXS data validate a two-dimer/DNA complex

**Fig. 5:**
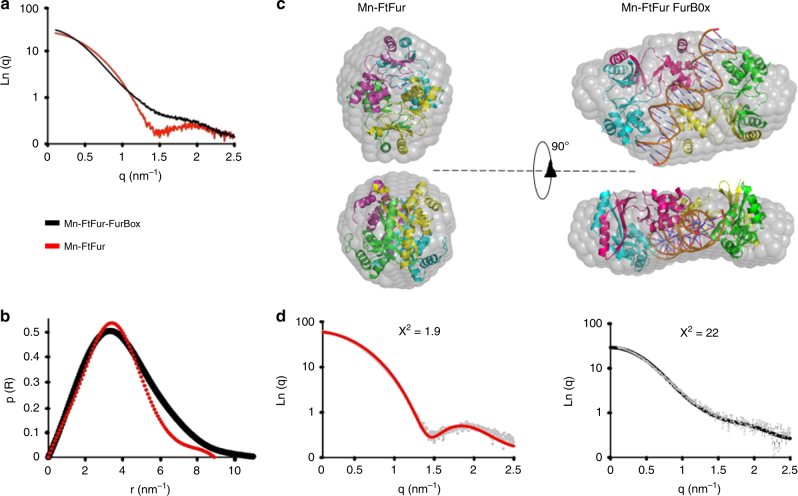
Comparison of small-angle X-ray scattering curves of Mn-FtFur and Mn-FtFur/FurBox complex in solution. **a** Average scattering curves of Mn-FtFur and Mn-FtFur/FurBox complex in solution by small-angle X-ray scattering. **b** Pair distance distribution functions, p(R): Mn-FtFur/FurBox (MW = 81 kDa) and Mn-FtFur (MW = 64 kDa). **c** Molecular models of Mn-FtFur structure (left) and of Mn-FtFur/FurBox (right) fitted in the SAXS envelope. The model of the Mn-FtFur/FurBox complex is obtained, in the absence of X-ray structure, by superposition of Mn-FtFur on the Mn-MgFur/FurBox complex (see Supplementary information). **d** Fits of the scattering curves

The activated Mn-bound FtFur form and the Mn-bound FtFur/EcFurBox complex were examined by small-angle X-ray scattering (SAXS). In both cases, at three different concentrations (1–10 mg mL^−1^), the linearity of the Guinier plots indicates monodisperse samples. Average scattering curves of Mn-FtFur (red) and Mn-FtFur/FurBox complex (black) in solution were recorded (Fig. 5a). Pair distance distribution functions (Fig. 5b) point out an elongation of the protein/DNA system (Dmax = 112 Å for DNA/protein complex against 83 Å for the protein alone) and dramatic changes in the shape of the structure (Porod volume = 130 nm^3^, against 100 nm^3^, and radius of gyration = 32.5 Å, against 27.4 Å). Bead molecular models of Mn-FtFur alone and in complex with DNA complex, built by DAMMIF^32^, show a globular Mn-FtFur and a thick pancake shape for the DNA complex (Fig. 5c). The X-ray structure of the protein determined in this study docks very well in the calculated envelope with a *χ*^2^ of 1.9 (Fig. 5d). In the absence of high-resolution structure of the Mn-FtFur–DNA complex, a model was built based on Mn-MgFur/EcFurBox ternary complex^29^ and fits well with the calculated envelope with a *χ*^2^ of 2.2. These results are in agreement with the conclusions of the cross-linking experiments and support the DNA-driven split of the FtFur tetramer in two dimers sandwiching the FurBox. To better understand the mechanism of dimer–dimer and dimer–DNA dissociation, theoretical calculations were performed.

### Dimer/dimer and dimer/DNA dissociation free energy profiles

**Fig. 6:**
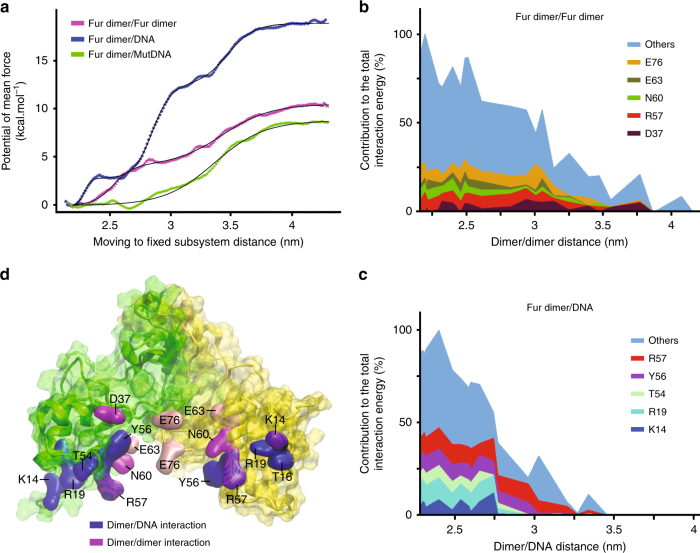
Computation of free energy profiles for dimer/dimer and dimer/DNA dissociation. **a** Potentials of mean force for the extraction of FtFur dimer from the tetramer or from DNA. The *x*-axis, reaction coordinate, corresponds to the average centre of mass/centre of mass distance between the 'fixed' and the 'moving' subsystems. Data points corresponding to the outputs of Wham are fitted with 1, 2 or 3 sigmoid functions with R^55^. Statistical errors were estimated using the bootstrap method. **b** Major contributors to the average interaction energy between Fur chain D and the 'fixed' dimer in the tetramer simulation. On average, five residues contribute to around 30% of the total interaction energy. **c** Major contributors to the average interaction energy between Fur chain D and DNA in the FtFur/wild-type DNA simulation. The *x*-axis corresponds to the average centre of mass/centre of mass distance between Fur and DNA. On average, five residues contribute to 54.5% of the total interaction energy. **d** Structure of FtFur dimer showing chain A (yellow) and chain D (green). Residues shown in blue surface are the major contributors to the average interaction energy between FtFur and DNA in the FtFur/wild-type DNA simulation. Residues in magenta and pink surfaces are the major contributors to the interaction energy between the 'moving' dimer and the 'fixed' dimer in the FtFur tetramer simulation. The mutated residues E63 and E76 are in pink colour

The aim of this modelling was to evaluate precisely the difference in binding affinity between the FtFur dimers within the FtFur tetramers and between the FtFur dimers and DNA. Free energy (potential of mean force) profiles for the extraction (by translation along a fixed direction: Ox) of one FtFur dimer from the tetramer (dimer of dimers) and of FtFur from DNA were computed: the meticulous translation protocol is shown in Supplementary Fig. 6. The simulations include a 'moving' subsystem (FtFur dimer, chains A and D) and a 'fixed' subsystem (FtFur dimer, chains B and C, DNA) as shown in Supplementary Fig. 7. The profiles were built using the 'umbrella sampling' technique and result from the overlapping of 26 computation windows, one for each translation distance, and corresponding to 15 ns molecular dynamics simulation each. The results of the calculations are shown in Fig. 6a. Binding free energies are Δ*G* = 18.8, 10.5 and 8.8 kcal mol^−1^ for dimer from FurBox, dimer from tetramer and dimer from mutated DNA (mutDNA containing FurBox_m_), respectively. These binding free energies correspond to dissociation constants of 17 fM, 20 nM and 0.4 µM, respectively, allowing a thermodynamically easy separation of the tetramer into two dimers in the close proximity of DNA, deduced from the experiments. Statistical errors were estimated to be <1.5 kcal mol^−1^ with bootstrap analysis using the 'Bayesian bootstrap' method (b-hist option in g_wham).

According to Fig. 6b and Supplementary Fig. 8A, the residues mainly contributing to the stability of the tetramer are: E76, E63, N60, R57, D37 and K14, in agreement with the experimental results where the mutation of residues E76 and E63 into alanine leads to easier dissociation of the FtFur tetramer into two dimers. Close inspection shows that R57 interacts with E63, E76 with N60 and D37 with S35. For both A and D moving chains, these residues contribute to around 30% of the total interaction energy.

The residues with the strongest contribution to the FtFur/wtDNA complex stability are R57, Y56, T54, R19, T16 and K14, contributing more than 50% of the total interaction energy (Fig. 6c and Supplementary Fig. 8D). By homology with the *Magnetospirillum gryphiswaldense* (Mg) Fur-DNA structure^29^, R57, Y56 and K14 are expected to make base-specific contacts, whereas T54, R19 and T16 interact with phosphates. Average interaction energy profiles between the 'moving' and 'fixed' subsystems are shown in Supplementary information. According to these profiles, the dissociation of the FtFur dimers from DNA would occur in two steps: slight unbinding of subunit D followed by unbinding of subunit A (Supplementary Fig. 8B, up and down). Noticeably, the mutations in the DNA FurBox drastically impede the binding of Fur to DNA with a 10 kcal mol^−1^ binding free energy decrease, explaining the selectivity of the binding of Fur to its FurBox sequence. More precisely, three of the four mutations face Fur chain D and their impact on the complex dissociation is visible in Supplementary Figure 8B where the initial average interaction energy of FtFur chain D with mutated DNA (−100 kcal mol^−1^) is around half that with WT DNA (−200 kcal mol^−1^). Below 2.8 nm the interaction of chain D with WT DNA remains stronger than with mutated DNA. (Supplementary Fig. 8C).

A summary of the FtFur dimer structure and the residues involved in its interactions with the other dimer within the tetramer or with DNA is shown in Fig. 6d.

### New FtFur regulation mechanism from models and mutation data

**Fig. 7:**
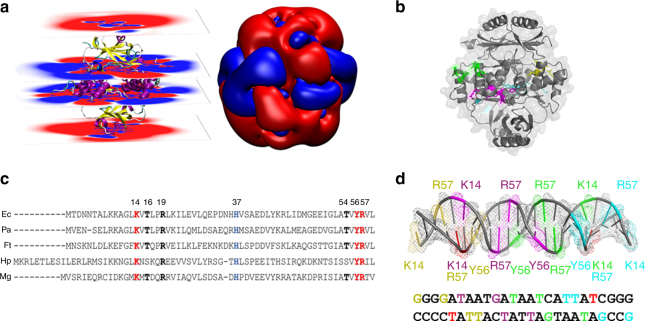
DNA-driven FtFur tetramer dissociation mechanism. **a** Electrostatic potential around FtFur calculated on parallel planes (left) and on equipotential surfaces at −0.1 (red) and 0.1 V (blue) (right). **b** Mn-FtFur structure with its solvent accessibility surface. The residues predicted to be involved in the DNA interaction are coloured. **c** Sequence alignment of the DNA-binding domains of five Fur proteins with known structure EcFur (DNA-binding domain X-ray structures only)^56^; PaFur^27^; FtFur (this work); HpFur (*Helicobacter pylori*)^22^ and MgFur^29^. The highly conserved amino acids implicated, in site S2 (blue) and in the interactions with DNA are in bold, coloured in red for those forming base-specific interactions and black for those having interactions with the phosphates (as evidenced in the structure of MgFur in complex with DNA). **d** Sketch of the DNA FurBox double-strand highlighting interactions with four Fur subunits (forming two dimers). Each of them is shown in a specific colour: yellow, purple, green and cyan, corresponding to the residues shown in **b**. Interactions between DNA bases and Fur residues are deduced from our results and the structures of the MgFur–DNA complex (T highlighted in red interact with two subunits)

Structural analysis suggests that the Arg57–Glu63 interaction plays a key role in the tetramer stabilization. Two of the four Arg57 (1 per dimer) are involved in such salt bridges and the two others are accessible to the solvent or to the DNA. Arg57 is predicted to be one of the most important residues for the interaction between Fur and bases in the specific DNA FurBox (Figs. 6, 7). This residue is highly conserved and its importance is in accordance with the Fur-DNA X-ray structure in *M. gryphiswaldense* where only few residues form base-specific interactions: Arg57-G7, Lys15-A24′ and Tyr56-T15′/T16′^29^. Similarly, Arg65 in EcZur, a Fur-like protein, interacts with a purine DNA base^30^. In FtFur, the four Lys14 (eq. Lys15 in MgFur) and two Arg57 are accessible for DNA interaction. Interestingly, the electrostatic potential around the tetramer shows a clear positive crown in the region of these residues (Fig. 7a) where the negatively charged DNA would be expected to first interact. We hypothesize that the specificity of the DNA-dependent tetramer dissociation could result from the interaction of DNA with Lys14 and the accessible Arg57, which would destabilize the tetramer by a progressive loss of the interaction of the two other Arg57 with Glu63. Mutations of Glu63 and/or Glu76 to Ala confirmed the importance of these residues in the stability of the tetramer since dimeric forms were obtained, partially for the single mutants and completely for the double mutant at high salt concentrations (Supplementary Fig. 5A, B). Both single mutants were still able to bind DNA in the presence of metal ions contrarily to the double mutant. Moreover, the Arg57–Glu63 salt bridge disruption should leave room to interactions of the crucial Tyr56 with DNA.

### Fur is involved in *F. tularensis* virulence and pathogenicity

The critical role of Fur in pathogenicity and virulence of several pathogens is known^16,33^. To investigate the putative role of FtFur as a virulence factor we compared the phenotypes of CHUGA-Ft6 and CHUGA-Ft6∆*fur* using in vitro or in vivo infection models. Three types of experiments were conducted: bacterial multiplication in J774-A1 murine macrophage-like cells, H_2_O_2_ sensitivity assay and in vivo virulence assays in mice.

**Fig. 8:**
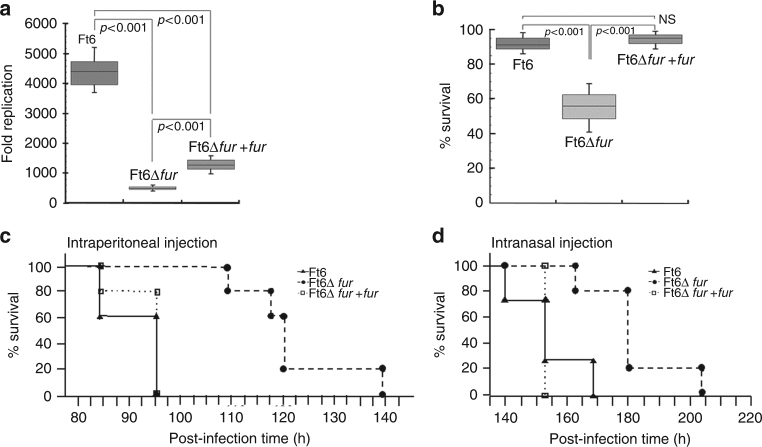
Fur is directly involved in *F. tularensis* virulence and pathogenicity. **a** Bacterial multiplication in J774-A1 murine macrophage-like cells. The data correspond to two independent experiments made in triplicate. *P* values were calculated using the Student's *t* test. **b** Hydrogen peroxide sensitivity assay expressed as the percent survival of each strain exposed 4 h to 1 mM H_2_O_2_. Bacterial suspensions were incubated 4 h with 1 mM H_2_O_2_ before enumeration of the CFU onto chocolate agar plates. Data are expressed as mean ± SEM of three distinct experiments. The data correspond to two independent experiments made in triplicate. *P* values were calculated using the Student's *t* test. **c**, **d** In vivo virulence assay in mice inoculated IP with 5e2 CFU in 500 µL of physiological serum or IN with 2e3 CFU in 50 µL of physiological serum. Five mice were used for each condition and experiments were performed twice. Survival curves were compared using the Kaplan–Meier test. *P* values were <0.001 for CHUGA-Ft6 and CHUGA-Ft6∆*fur* + *fur* vs. CHUGA-Ft6∆*fur*, for both the IP and IN routes. No significant difference was found between CHUGA-Ft6 and CHUGA-Ft6∆*fur* + *fur*

A growth defect of the CHUGA-Ft6 mutant lacking *fur* in liquid medium was evidenced as shown by a longer lag time, a longer generation time and a lower optical density at the stationary phase as compared to the WT parental strain (Supplementary Fig. 1B). A similar phenotype was observed on solid medium with a delayed onset of visible colonies and a smaller size of colonies for CHUGA-Ft6∆*fur* (Supplementary Fig. 1C). The ability of CHUGA-Ft6, CHUGA-Ft6∆*fur* and CHUGA-Ft6∆*fur* + *fur* to replicate within macrophages was then evaluated by infecting J774-A1 murine macrophage-like cells. One hour after infection, the host cells contained the same number of intracellular bacteria regardless of the infecting strain meaning that Fur is not required for macrophage infection. After 24 h incubation (Fig. 8a), the number of intracellular bacteria was markedly different as the WT cells were eight-fold more abundant compared to the CHUGA-Ft6∆*fur*. The *fur*-complemented strain showed an intermediate level of intracellular macrophage multiplication. The ability of these bacterial strains to resist an oxidative stress corresponding to the respiratory burst set up by infected macrophages was also checked by growing bacteria previously exposed to 1 mM H_2_O_2_ during 4 h (Fig. 8b). CHUGA-Ft6 and CHUGA-Ft6∆*fur* + *fur* displayed a similar percentage of survival while Ft6∆*fur* was much more sensitive to the oxidative stress with about 50% of surviving cells.

The involvement of *fur* in the infectious process in vivo was then evaluated by using mice infected with *F. tularensis* by intranasal (IN) or intraperitoneal (IP) administration (Fig. 8c, d). The survival curves of the animals showed that regardless of the administration route, CHUGA-Ft6 and CHUGA-Ft6∆*fur* + *fur* caused the mice death in approximately the same delay, which is 95 h post-infection for IP and 150 to 168 h for IN inoculation. On the other hand, mice infected with CHUGA-Ft6∆*fur* survived a significantly longer time (*p* < 0.001 compared to CHUGA-Ft6 and CHUGA-Ft6∆*fur* + *fur* whatever the route of infection), that is, 140 h and more than 200 h for the last animal infected by IP and by IN routes, respectively. These results define *fur* as an important virulence-associated gene in *F. tularensis* and are a further example that deletion of this gene leads to an attenuated phenotype in terms of virulence.

## Discussion

Altogether, the involvement of Fur in the iron homeostasis and the virulence of *F. tularensis* have been demonstrated here as well as its direct interaction with the *figA* promoter region. FtFur belongs to the new family of tetrameric Fur proteins. It contains the structural zinc site S1 and the regulatory site S2 and lacks the third site S3, usually found in Fur proteins. S1 is not present in tetrameric PaFur and S3 is absent in FtFur, which still forms a tetramer upon deletion of S2, indicating that the tetrameric state of the protein does not rely on such sites. To our knowledge, the first published structure of FtFur containing the physiologically relevant ferrous iron is presented here with a ferrous ion in an octahedral geometry. Metalled FtFur behaves as a pre-activated tetramer with the DNA-binding domains forming a positively charged crown where the recognition helices interact through two stabilizing salt bridges between two Arg57 (out of four) and two Glu63. The tetramer dissociation is driven by an interaction of the protein with a specific DNA sequence, suggesting the involvement of the two free Arg57 and Lys14, known to form base-specific contacts with DNA. We postulate that the two H-bound Arg57 would progressively lose their interaction with Glu63 replaced by interactions with DNA, leading to the breaking of the salt bridge, crucial for the stability of the tetramer and its dissociation into two dimers specifically bound to the FurBox. In vivo studies reveal that FtFur is important for the virulence of *F. tularensis*. Because there is no efficient vaccine and only few poorly efficient antibiotics available to fight tularaemia, this work shows that Fur is an attractive anti-virulence target for new inhibitors, whose design, starting from already known inhibitors against other Fur proteins^34^, will be facilitated by the detailed structure and mechanism of interaction with DNA.

## Methods

### Bacterial strains and culture media

The biovar I strain of *F. tularensis* subsp. *holarctica* used in the virulence assay, referred as CHUGA-Ft6, was isolated at Verdun Hospital (France) from a blood sample collected during routine care of a patient with typhoidal tularaemia. Identification at the species and subspecies level was obtained by PCR amplification and sequencing of the intergenic region between the 16SrRNA and 23SrRNA encoding genes^35^. Bacterial cultures were performed either on chocolate agar plates supplemented with PolyVitex (CPV, Biomérieux, Lyon, France) or in liquid brain heart infusion medium supplemented with 2% PolyViteX (BHI-2%PV). When necessary, kanamycin (10 µg mL^−1^) or sucrose (5% (w/v)) was added. Cultures were incubated at 37 °C, in a 5% CO_2_-enriched atmosphere. Intracellular iron concentration was measured on stationary phase bacteria grown over 15 h in modified Mueller–Hinton medium into a shaking incubator (200 rpm at 37 °C). Briefly, the cells have been washed several times with phosphate-buffered saline-ethylenediaminetetraacetic acid (PBS-EDTA) 10 mM before hydrolysis with HNO_3_ at 65% ON at 95 °C and measurements by ICP-AES (Shimadzu ICP 9000 instrument with Mini plasma Torch in axial reading mode^20^).

### Construction and complementation of the Ft6 Δ*fur* mutant

A phase deletion of the *fur* gene was carried out in the CHUGA-Ft6 virulent isolate, by the method of allelic exchange (Supplementary Fig. 1), through the use of a suicide plasmid containing the *sacB* gene, pMP812^36^. Approximately 1000 bp adjacent regions of the Ft6 *fur* gene were amplified using the two primers LeftfurF-LeftfurR and RightfurF-RightfurR (Supplementary Table 4). The obtained PCR products were mixed and further submitted to a second PCR using the forward primer LeftfurF and the reverse primer RightfurR, generating a PCR fragment containing the two adjacent regions of *fur* flanked with the BamHI and EcoRV restriction sites. This fragment was digested with the two corresponding restriction enzymes and cloned into the plasmid pMP812^36^ previously digested with the same enzymes. After electroporation, several selection (kanamycin, then sucrose) steps were performed to obtain a delta *fur* mutant devoid of antibiotic resistance. In order to complement the strain Ft6Δ*fur*, the *fur* gene and its promoter region were amplified using CompfurL and CompfurR primers and cloned the shuttle vector pMP828. The plasmid pMP828 containing the *fur* gene was then electropored into CHUGA-Ft6Δ*fur* and complemented colonies selected on agar plates supplemented with kanamycin. The *fur* expression in resulting transformants was checked using a specific qRT-PCR.

### Evaluation of the gene expression by qRT-PCR

Gene expression was measured from strains grown for 16 h in BHI-2%PV. Approximately 10^7^ cells were collected for total RNA extraction that was achieved using 1 mL TRIzol^®^ reagent (Invitrogen) and following the manufacturer’s instructions. Contaminating DNA was removed using the TURBO DNA-free™ Kit (Ambion, Life Technology). The first*-*strand complementary DNA (cDNA) synthesis reaction was carried out starting from 500 ng of purified RNA and using the SuperScript™ II Reverse Transcriptase Kit (Invitrogen, Life Technology). The resulting cDNA library was used as a template in combination with the specific primers for qRT-PCR, which was conducted using a Fast SYBR Green MasterMix in a StepOnePlus Real Time PCR Systems (Applied Biosystems). Cycling was 20 s at 95°C; 3 s at 95°C, 30 s at 60 °C, repeated for 40 cycles. The expression level of each target gene was calculated from three independent experiments and expressed as a ratio taking the expression of the housekeeping gene 16S RNA as the denominator. Primer sequences are indicated in Supplementary Table 5.

### DNA sample preparation

DNA oligonucleotides were synthesized by MWG at high purity scale. DNA duplexes were first annealed in water at concentration of 20 mg mL^−1^ by heating the mixture at 95 °C for 5 min and rapid cooling on ice in buffer A (50 mM Tris-HCl, pH 8.8, 150 mM NaCl) and then stored at 4 °C. The formation and concentration of DNA duplexes were determined by SEC-MALLS-RI in binding buffer. DNA was used extemporary for biochemical experiment.

### Protein expression and purification

#### Apo-FtFurWT

Recombinant FtFur of *F. tularensis* FSC198 ref: NC-008245.1, 100% identical in sequence to the CHUGA-Ft6 protein, was purified as a tetramer containing one equivalent of Zn per subunit, as previously described^20^. It was over-produced in BL21 DE3 R2 *E. coli* strain in Luria–Bertani (LB) medium after an overnight induction with 1 mM isopropyl β-d-1-thiogalactopyranoside (IPTG) at 18 °C. Cell were resuspended in buffer A, lysed by sonication and purified successively on several columns (GE Healthcare): (1) ion-exchange chromatography on DEAE Sepharose with linear gradient between buffer A and buffer A supplemented with 1 M NaCl, (2) hydrophobic interaction chromatography on Butyl fast flow Sepharose with linear gradient between buffer A containing 1 M of ammonium sulphate and buffer A and (3) size-exclusion chromatography Superdex-200 (10/60) equilibrated with buffer A.

#### Apo-FtFur mutants

E63A, E76A and E63A-E76A mutants were cloned in pET-TEV (based on pET28a) vector to produce N-terminal 6×HisTag cleavable TEV fusion proteins. PCR was done in the presence of appropriate primer with Phusion polymerase-HF at recommended *T*_m_. PCR samples were incubated with a reaction buffer containing 2 UI of *Dpn*I, 10 UI of T4 DNA ligase, 1 mM of ATP and 2 UI polynucleotide kinase (PNK) in PNK buffer from NEB for 15 min at room temperature (RT) before transformation in Top10 ultracompetent cells. Each mutant has been DNA-sequenced before expression and purification as described. H33A-H90A double mutant (FtFurΔS2) was obtained from pET30b-FtFurWT before cloning in pET-TEV, over-produced and purified like FtFurWT. The other mutants were over-produced in in BL21 (DE3) R2 *E. coli* strain LB medium after induction with 1 mM IPTG at 37 °C for 4 h. Purification was done by using Ni-NTA resin (Qiagen) in batch mode in buffer A with 10 mM imidazole and 10% glycerol. Pure protein fractions were pooled and mixed with homemade HisTag-TEV protease (1% of the protein concentration to purify) together with 1 mM dithiothreitol (DTT) and 1 mM EDTA. The solution was then dialysed using a 3 kDa cut-off membrane against 2 L buffer A containing 1 mM DTT and 1 mM EDTA at 4 °C overnight, followed by a second 2 L dialysis against buffer A to remove DTT and EDTA. The protein sample was then passed through the Ni-NTA column in order to separate the pure protein from the HisTag-TEV protein and the HisTag itself. A final step of purification was performed by using Superdex-200 in buffer A supplemented with 500 mM NaCl at 4 °C. Collected fractions were concentrated on a 50 kDa cut-off Vivaspin from 20 to 40 mg mL^−1^ and used or frozen in liquid nitrogen in the presence of 10% of glycerol before storage at −80 °C.

#### Purification of the Mn-FtFur/Fur Box complex

The purification was done in buffer B (50 mM HEPES, pH 7.5, 150 mM NaCl, 1 mM MnCl_2_, 1 mM MgCl_2_) at 20 °C. Molar equivalents (1.2) of FurBox Duplex (see DNA sample preparation) were incubated with Mn-FtFur before loading on a Superdex-200 increase 10/30 GE Healthcare column equilibrated with buffer B. Pooled fractions were analysed in SEC-MALLS-RI in the same buffer to check the integrity of the complex.

### EMSA and nuclease activity assay

EMSA experiments were performed as previously described^20^. The formation of small-scale (under 1 µg) DNA duplexes was confirmed by native gel electrophoresis^20^ on 10% acrylamide gel in 1× TAE buffer (40 mM Tris-acetate, pH 8.2, 1 mM EDTA). DNA radiolabelling was performed by incubating 20 nM DNA for 30 min at 37 °C in the presence of 1 U of T4 polynucleotide kinase (NEB) and 1 µL of γATP at 1 mCi mmol^−1^. Labelled DNA was diluted 10 times in buffer A, desalted on G25 Mini Spin Column and stored at −20 °C. EMSA were performed with 250 pM of freshly prepared radiolabelled DNA incubated 30 min at 25 °C with different concentrations of protein in a binding buffer (20 mM Bis-Tris propane, pH 8.5, 100 mM KCl, 3 mM MgCl_2_, 10 µM MnCl_2_, 5% (v/v) glycerol and 0.01% Triton X-100). After 30 min incubation at room temperature, 10 µL of each sample were loaded on 10 % polyacrylamide (29/1) gel. The gel was pre-run for 30 min at 100 V in TA buffer (40 mM Tris-acetate, pH 8.2) supplemented with 100 µM of MnCl_2_. Mobility shifts were revealed by exposing the gels on a storage phosphor screen (GE Healthcare) and quantified with a cyclone phosphoimager (Perkin Elmer). The nuclease activity assay was performed as previously described^20^.

### SEC-MALLS-RI experiments

Each sample was checked by size-exclusion chromatography coupled to multi-angle laser light scattering with online refractive index (SEC-MALLS-RI) as previously described^20^ and using a standard procedure: 20 µL of sample with a 2 to 10 mg mL^−1^ concentration were loaded on an analytical Superdex-S200 increase pre-equilibrated at 0.5 mL min^−1^ with appropriate buffer (Apo-FtFur in buffer A, Mn-FtFur and Mn-FtFur/FurBox in 50 mM HEPES, pH 7.5, 150 mM NaCl, 1 mM MnCl_2_, 1 mM MgCl_2_) and connected to an in-line DAWN HELEOS II spectrometer (Wyatt Instruments). An in-line refractive index detector (Optirex, Wyatt Instruments) was used to follow the differential refractive index relative to the solvent. After baseline subtraction of the buffer solution, all samples presented a single peak allowing the determination of absolute molecular masses with the Debye model using ASTRA6 software (Wyatt Instruments) and a theoretical dn/dc value of 0.185 mL g^−1^. The final values correspond to the average of three independent experiments.

### Crystallization

Protein crystallization conditions were obtained by using crystallization screens (Hampton Resarch Grid Screens^TM^ and Qiagen protein crystallization suites) with the HTX Lab high-throughput robot screening (HTX Lab at EMBL-Grenoble). Diffracting crystals up to 8 Å were obtained in 50 mM MES, pH 5.6, 200 mM KCl, 5% (w/v) PEG 8000, 10 mM magnesium chloride hexahydrate. This crystallization condition was then manually optimized. Diffracting crystals up to 1.7 Å were obtained by 1 µL of a 16 mg mL^−1^ Mn-FtFur holoprotein solution with 1 µL of 50 mM MES, pH 5.8, 20% (w/v) PEG 3350, 200 mM MgCl_2_·6H_2_O, 10 mM MnCl_2_ reservoir solution, using the hanging drop vapour-diffusion method. Crystals of Fe-FtFur holoproteins were obtained in the same condition in the presence of 10 mM of (NH_4_)_2_Fe(SO_4_)_2_·6 H_2_O under anaerobic condition in a glove box. Crystals appeared in a few days and were back-soaked three successive times in a mother liquor containing 50 mM MES, pH 5.8, 20% (w/v) PEG 3350, 200 mM MgCl_2_·6H_2_O, to remove the excess of free metal. All crystals were cryoprotected using a solution obtained by adding 25% (v/v) glycerol to the mother liquor containing 25% (w/v) PEG 3350 and flash-cooled in liquid nitrogen.

### X-ray diffraction and structure resolution of Mn-FtFur and Fe-FtFur

Diffraction experiments were done on the beamline FIP-BM30-ESRF (European Synchrotron Radiation Facility, Grenoble, France).

For Mn-FtFur, a fluorescence spectrum was recorded to check the presence of Mn at 1.77 Å (right side of maximum Fd″ for manganese) and Zn at 0.97 Å cations (Supplementary Fig. 3A). A remote and an anomalous datasets were recorded at wavelengths 0.97 and 1.77 Å (right side of maximum F″ for manganese). Best dataset (0.97 Å) diffracted at 1.7 Å resolution. Diffraction data were integrated/scaled in the space group P2_1_ using XDS Program Package version 15 October 2015^37^. The structure was solved by the SAD method using Phenix 1.10.1-2155/AutoSol^38^ and 86% of the model was built automatically. The model was rebuilt/corrected manually and refined using alternatively COOT^39^ and REFMAC^40,41^. Final refinement cycle was done in Phenix (Supplementary Table 1 and Supplementary Fig. 3B).

For Holo-Fe-FtFur, a fluorescence spectrum was recorded to check the presence of cations: Fe and Zn at 0.97 Å (Supplementary Fig. 3A). The datasets were collected at wavelengths 0.97 Å with a resolution of 1.8 Å, integrated/scale by XDS Program Package in the space group P2_1_.

The structure was solved by molecular replacement using MOLREP^42^ with the previous structure (Mn-FtFur) as the starting model. Indeed, we were not able to obtain a Fe-FtFur structure in the same conditions using iron Mohr salt in place of manganese even in a glove box. When trying to obtain the crystal from SEC-purified Mn-FtFur–DNA complex, only the same Mn-FtFur crystals were seen, but adding iron in the crystallization conditions, we were able to get crystals of Fe-FtFur diffracting at 1.8 Å and to solve the structure by molecular replacement with Mn-FtFur. The anomalous dataset was used to confirm the presence of Fe in the structure. The model was built and refined using REFMAC and COOT alternatively (Supplementary Table 2 and Supplementary Fig. 3B).

Both Holo-Mn-FtFur and Holo-Fe-FtFur final structures were validated by molprobity^43^. Protein Data Bank (PDB) redo^44^ was used before deposition of the structures to the PDB. The PDB codes are 5NBC for Holo-Mn-FtFur and 5NHK for Holo-Fe-FtFur. King software (in Phenix) was also used to cross-validate the data.

### SAXS experiments

Before each experiment, all samples were extemporaneously re-purified on SEC Superdex-200 increase (GE Healthcare) 10/300 equilibrated in an appropriate buffer. SAXS data were collected at the ESRF (Grenoble, France) on beamline BM29 BioSAXS. The scattering profiles were measured at several concentrations between 0.5 to more than 10 mg mL^−1^. Data were processed using standard procedures with the ATSAS v2.5.1 suite of programs^45^ as described^20^. The ab initio determination of the molecular shape of the proteins was done as previously described^20^. Briefly, radius of gyration (*R*_g_), forward intensity at zero angle (*I*(0)), Porod volumes and Kratky plot were determined using the Guinier approximation and PRIMUS programs^46^. In order to build ab initio models, several independent DAMMIF^32^ models were calculated in slow mode with pseudo-chain option and analysed using the program DAMAVER^47^. Docking of the tetrameric X-ray structure into the measured SAXS envelope was generated by SUPCOMB^48^. The model of the Mn-FtFur/FurBox complex was built from the Mn-MgFur/FurBox structure (PDB code 4RB1): after sequence alignment, the atom coordinates of the corresponding amino acids were directly copied from MgFur to FtFur and coordinates of missing side chain atoms were added from internal coordinates. The resulting model was energy minimized with CHARMM^49^. The program CRYSOL^32^ was used to generate the theoretical scattering curves from the tetrameric structure of FtFur.

### Cross-linking experiments

Cross-linking experiments between FtFur and Fur boxes were performed using 0.1% GTA. With a short spacer arm of approx: 5 Å, and when used at a low concentration, this cross-linker agent is well suited for intramolecular cross-linking and to specifically cross-link individual species in close interactions. Two micrograms of Mn-FtFur were used in each tube, fresh GTA was used at 0.1% and 25–50 and 100 ng of DNA oligonucleotides duplex was added sequentially. Incubation buffer are done with 1 mM of fresh MnCl_2_ at RT before being cross-linked by GTA 30 min at RT and loaded onto sodium dodecyl sulphate-polyacrylamide gel electrophoresis 4–20% acrylamide gradient.

### Construction of the models

The X-ray structure of FtFur solved in this study (Asp7 to Arg137) was used as the initial model for the tetramer. The GROMACS program version 5.1.2^50^ with the gromos54a7 united atom force field^51^ was used to perform long molecular dynamics simulations needed to compute free energy profiles. Fe^2+^ and Zn^2+^ were modelled as simple Lennard Jones hard spheres with charge +2 with Zn coordinated to charged deprotonated cysteines (see for details Supplementary Methods).

In the absence of FtFur + DNA structure (FtFur/wtDNA), the structure of *M. gryphiswaldense* (4RB1)^29^ in the presence of DNA was used to model the wtDNA FurBox and correctly position FtFur dimer on wtDNA (by least-square fit matching of atom positions). The 5′-GCCG**GATAATGATAATCATTATC**-3′ fragment (consensus FurBox in bold) and its complementary 3′–5′ sequence was used to model double-stranded wtDNA.

The mutDNA (equivalent to FurBox_m_) sequence GCCGGATA**C**TGATA**G**TC**C**T**G**ATC contains four mutations with respect to the FurBox (A9 to C, A15 to G, A18 to C and T20 to G; see nucleotides set in bold font) which were constructed by simple matching of corresponding heavy atoms in the WT DNA model and building of missing hydrogens. The three above vacuum systems were immersed in parallelepipedic SPC^52^ water boxes modelled with periodic boundary conditions after the addition of Na^+^ and Cl^–^ counterions to ensure neutrality and a total ionic force of 0.1 mol L^−1^. The solvated systems were energy minimized and equilibrated under NPT (constant Number of particles, Pressure and Temperature) conditions at 310 K and 1 atm.

### Computation of free energy profiles

Free energy profiles for the extraction (by translation along a fixed direction: Ox) of one FtFur dimer from the tetramer (dimer of dimers) and of FtFur from DNA were computed: the meticulous translation protocol is shown in Supplementary Fig. 6.

The simulations include a 'moving' subsystem (FtFur dimer, chains A and D) and a 'fixed' subsystem (FtFur dimer, chains B and C, wtDNA or mutDNA) as shown in Supplementary Fig. 7.

The profiles were built using the 'umbrella sampling' technique and result from the overlapping of 26 computation windows, one for each translation distance.

Each window consisted of 100 ps NPT equilibration and 10 to 15 ns NPT production simulations. Position restraints on the 'fixed' subsystem and distance restraints on the whole protein, in the form of NOE-type restraints (nuclear Overhauser effect) between H-bonded H and O atoms to maintain its secondary structure, were applied. The 'moving' subsystem was subject to two harmonic biasing forces along the X direction only ('umbrella potential') applied between the centres of mass of the 2 Fur dimer subunits and the centre of mass of the 'fixed' subsystem.

After the dynamics runs, positions and forces were collected from the trajectories and the umbrella sampling harmonic potential was unbiased using the Wham algorithm^53^ implemented in the 'g_wham' program^54^ to yield the free energy profiles.

### Computation of average interaction energy profiles

Interaction energy profiles were computed by extracting nonbonded interactions (electrostatic + Lennard Jones potential energies) from all the trajectories of the simulations and averaging for each window. Interaction energies were calculated between each residue in the Fur 'moving' dimer (chains A and D) and the 'fixed' subsystem (DNA or 'fixed' FtFur dimer).

### H_2_O_2_ sensitivity assay

Three independent overnight cultures of each strain (CHUGA-Ft6, CHUGA-Ft6Δ*fur* and CHUGA-Ft6Δ*fur* *+* *fur*) in BHI-2%PV medium were diluted in PBS to obtain 1 × 10^7^ colony-forming unit (CFU) mL^−1^ inocula. These bacterial suspensions were incubated 4 h with 1 mM H_2_O_2_ at room temperature without shaking. Culture samples taken before and after H_2_O_2_ exposure were serially diluted and spread onto CPV plates and incubated for 3 days at 37 °C. Counting of CFUs allowed to determine the percentage of surviving bacteria after H_2_O_2_ exposure. Data were expressed as the mean of three independent experiments.

### In vitro macrophage infection

J774-A1 murine macrophage-like cells were grown in 24-well microplate using Dulbecco's modified Eagle's medium (DMEM) (Gibco) medium supplemented with 10% decomplemented fetal calf serum (FCS, Gibco) at 37 °C in 5% CO_2_ enriched atmosphere. For infections assays, confluent J774-A1 monolayers were infected with a bacterium inoculum at a multiplicity of infection of 10:1 (approximately 5 × 10^6^ bacteria for 5 × 10^5^ cells), and reincubated for 1 h (37 °C, 5% CO_2_). The cell monolayers were then washed with PBS (Gibco), and DMEM-10% FCS containing 5 µg mL^−1^ of gentamycin was added for 1 h to kill non-phagocytized bacteria. The cell supernatant was then replaced with MEM-10% FCS, and cell cultures were incubated for 24 h (37 °C, 5% CO_2_). To evaluate bacterial multiplication within J774-A1 macrophages, infected cell monolayers treated with 0.1 % saponin immediately after gentamycin treatment (T0) or after 24 h incubation (T24) were lysed and serially diluted in PBS and plated onto chocolate agar plates enriched with PolyVitex for CFU numeration. Each point was performed in triplicate. Data obtained were compared using Student’s *t* test and *P* values <0.05 were considered statistically significant.

### In vivo virulence assay

Six-week- to eight-week-old BALB/c males were infected with overnight cultures of the strains CHUGA-Ft6, CHUGA-Ft6Δ*fur* and CHUGA-Ft6Δ*fur* *+* *fur* diluted in 0.9% NaCl. Experiments were performed in an animal biosafety level 3 laboratory. For each bacterial strain, groups of five mice were inoculated either intraperitoneally (500 CFUs in 500 µL) or intranasally (2000 CFUs in 50 µL) and infected animals were monitored several times a day, weighed every day, and euthanized when they had reached one of the following limit point: prostration, high piloerection, weight loss >15% of T0 weight and closed eyes. A group of unifected mice was used as control. All murine experiments were approved by our local ethics committee (ComEth, Grenoble, France). During the experiments, mice were monitored several times a day, weighed every day and euthanized when we felt they had reached our estimated limit point (prostrate, high piloerection, weight loss >15% of T0 weight and eyes closed). All these experiments were performed in compliance with the laws and regulations regarding animal experimentation in France.

### Data availability

The datasets generated during the current study are available from the corresponding authors on reasonable request. Coordinates and structure factors for Mn-FtFur and Fe-FtFur have been deposited in the RCSB Protein Data Bank under accession codes 5NBC and 5NHK, respectively.

## Electronic supplementary material

Supplementary Information
