# Supplementary material for: Structural and functional studies of the metalloregulator Fur identify a promoter-binding mechanism and its role in Francisella tularensis virulence
Source: Commun Biol. 2018 Jul 17;1:93. doi: 10.1038/s42003-018-0095-6 (PMC6123631; doi:10.1038/s42003-018-0095-6)
Supplement: Supplementary file 1 — Supplementary Information [file 42003_2018_95_MOESM1_ESM.pdf]

Supplementary information

Supplementary Figures

A

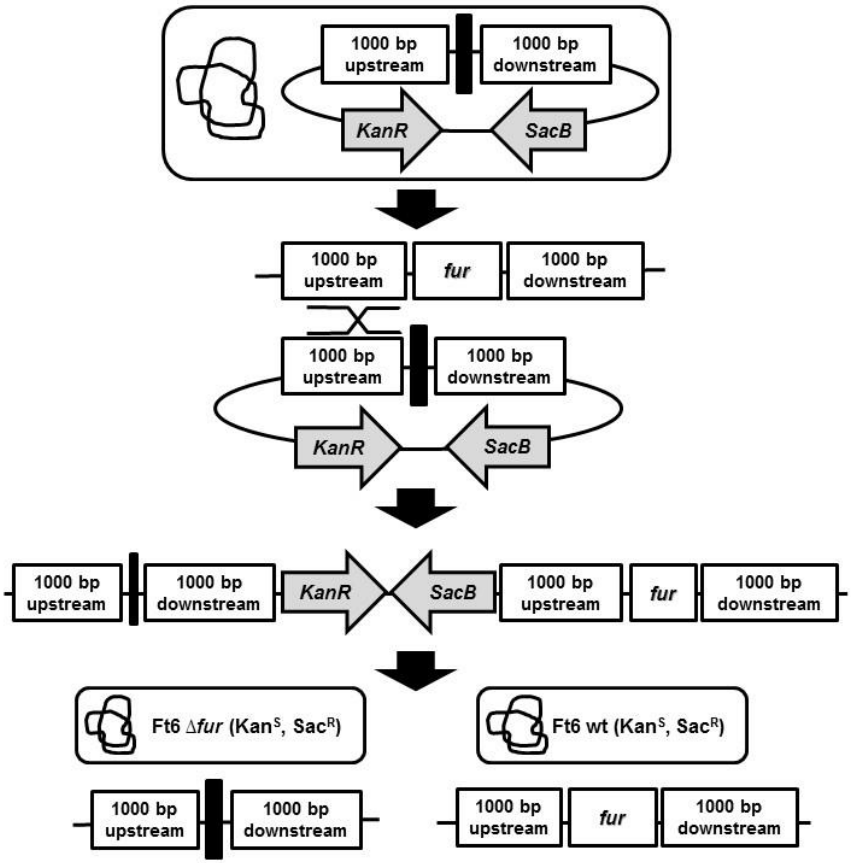

B

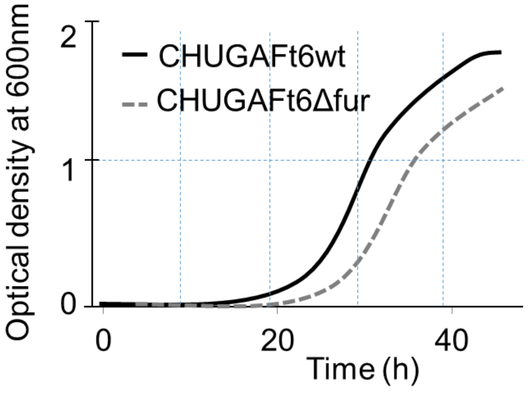

C

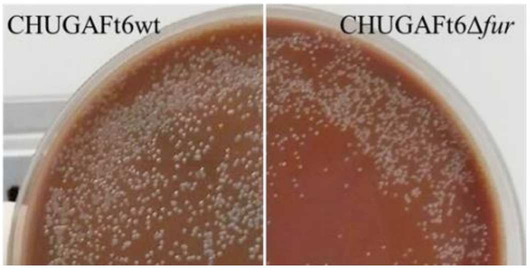

Supplementary Figure 1. Construction procedure of CHUGA-Ft6Δ*fur* and growth comparison with CHUGA-Ft6wt.

Panel A. Schematic representation of allelic change procedure. **Panel B.** Growth curve comparison of CHUGA-Ft6wt and CHUGA-Ft6Δ*fur* by monitoring the optical density at 600 nm as a function of time. **Panel C.** Colony morphology of CHUGA-Ft6wt and CHUGA-Ft6Δ*fur*. Bacteria grown for 72 h on Chocolate-agar plates (CPV) at 37°C with 5% CO_2 atmosphere.

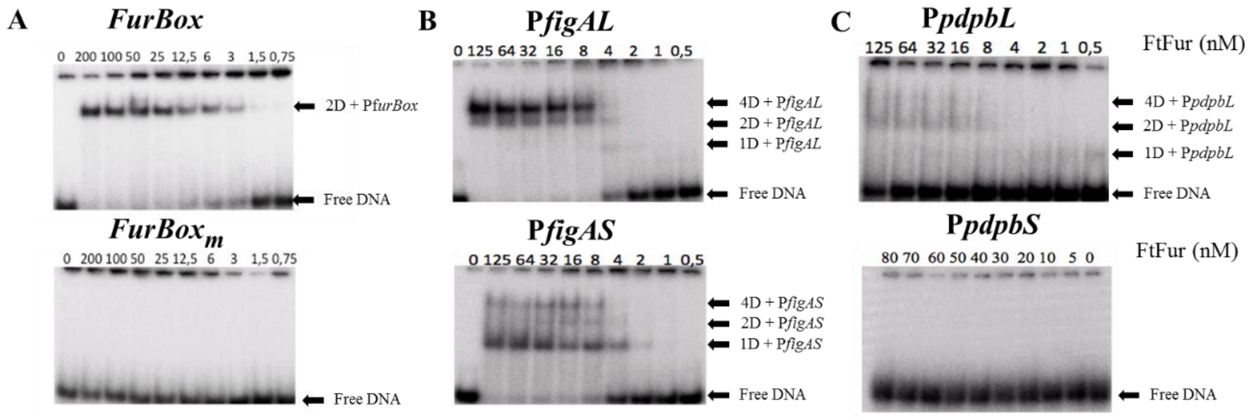

D

Name	Sequence
FurBox	GGGGATAATGATAATCATTATCGGG
FurBoxM	GGGGATACTGATAGTCCTGATCGGG
P*figAL*	GGGCTACATGATAATGATAACGAATATCATTATCGTTTATGGG
P*figAS*	GGGGATAATGATAACGAATATCATTATCGGG
P*pdpbL*	GGGCAAGTAAATGAAGATTGTGAGAATTCTTTTTTGATAAATGATAAAAAAAGGG
P*pdpbS*	GGGGATTGTGAGAATTCTTTTTGGG

E

```
PdpbL  CHUGA-Ft6
5' GGGCAAGTAAATGAAGATTGTGAGAATTCTTTTTTGATAAATGATAAAAAAAGGG 3'
         G--AATGA--AT--T-A--                              10/19
            -AT-A-GAT--T-A-AT-                            10/19
               GA--AT--T-A--ATT-T-                        10/19
                  GAT--TGA-AAT--TT-T-                     12/19
                                --T--TGATAA----TA--       9/19
iglC Schu S4
        GGGGTACAAATAATAACTAAAGGG
          G-TA---ATAAT-A-TA--   11/19

figA Schu S4
5' ATACTTAAATAAACGATAATGATATTCGTTATCATTATCATGTAGGGATT 3'
           ---AA-GATAAT-AT--TC                            12/19
                 GATAATGATA-TC-TTATC                      17/19
                       GATA-T--T-ATCATTATC                15/19
                             --T-AT-AT-ATCAT----          10/19
```

F

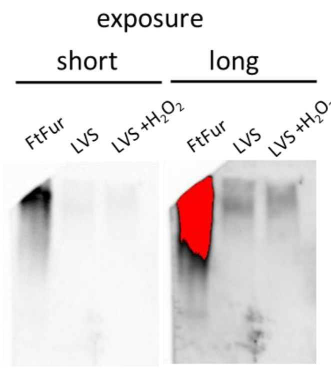

same migration

G

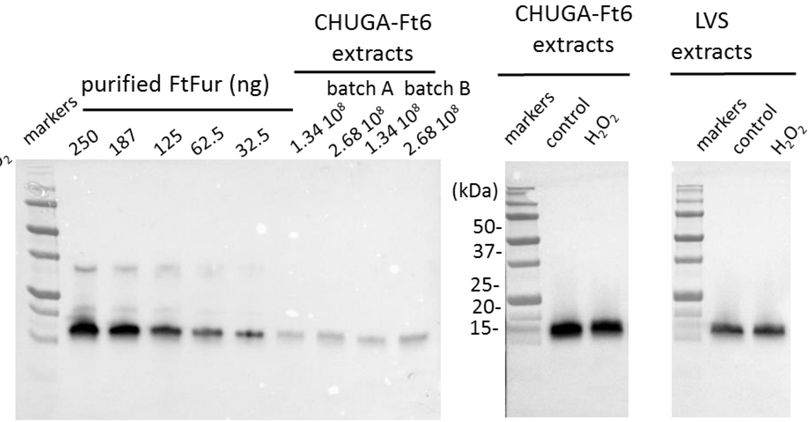

3000 subunits /CHUGA-Ft6 bacteria

	control	1 mM H_2O_2
**CHUGA-Ft6**	100%	93 % ±4
**LVS**	100%	105 % ±8

Supplementary Figure 2. DNA binding experiment by EMSA of FurBox containing promoters identified in *Francisella tularensis* genome and Western blot assays.

Panel A. FurBox corresponding to consensus sequence and a mutated FurBox as control ($FurBox_m$) previously described[4].
Panel B. P*fig*AL(long) and P*fig*AS (short) correspond to the genomic DNA sequence of CHUGA-Ft6 *Francisella tularensis*. The long version is already presented in Fig. 1 in the main text, the short version presents a different profile, with a major band corresponding to 1 dimer. DNA sequence analysis indicates the presence of 3 modifications compared to the FurBox, which could be important to the binding of FtFur on DNA.
Panel C. Result obtained with P*pdpb*L and P*pdpb*S are presented and indicate the low affinity of FtFur for this box. DNA sequence analysis indicates the presence of 6 modifications in nucleotide sequence and validates the low affinity obtained with this promoter.
Panel D. Sequence of forward primer used for EMSA experiment. The nucleotides corresponding to the consensus FurBox are underlined.
Panel E. promoters containing FurBox (with the homology with the consensus 19bp) are identified in *Francisella tularensis* genomes.
Panel F. Native Western blot on PAGE of *F. tularensis* live vaccine strain (LVS) extracts. For safety reasons, we are not allowed to manipulate the clinical CHUGA-Ft6 strain extracts outside the P3 laboratory without a previous denaturation step. We thus used LVS which contains an identical FtFur protein. Exponential growth phase bacteria were or not exposed for 4 h to 1 mM H_2O_2 at 37°C before lysis with BugBuster extraction reagent (Sigma). The recombinant purified FtFur (2.5 µg) and LVS extracts (10 µg) were loaded onto a 6% acrylamide gel and separated under non-denaturing conditions (100 V, 2 h). After transfer on nitrocellulose membrane, the samples were probed with anti-Ec Fur antibodies (1:15,000) and Fur was detected with HRP-conjugated goat anti-rabbit IgG antibody (Jackson Immunoresearch 1: 10,000) and visualized by chemiluminescence with the Biorad ECL substrate and using the Biorad chemidoc MP imaging system.
Panel G. Left panel: Quantification of the Fur proteins by the signal analysis obtained starting from various amount of recombinant protein and bacteria and leading to an average estimation of 3,000 Fur subunits per CHUGA-Ft6 bacteria. **Right** panel: Western blot of whole bacterial extracts of bacteria LVS and CHUGA-Ft6 grown in Modified Mueller Hinton medium supplemented with 0.0025 % ferric pyrophosphate and treated or not with H_2O_2 (1 mM for 4 h). Bacterial pellets resuspended in Laemmli buffer were separated by SDS-PAGE (14% acrylamide) and then processed as above.

A

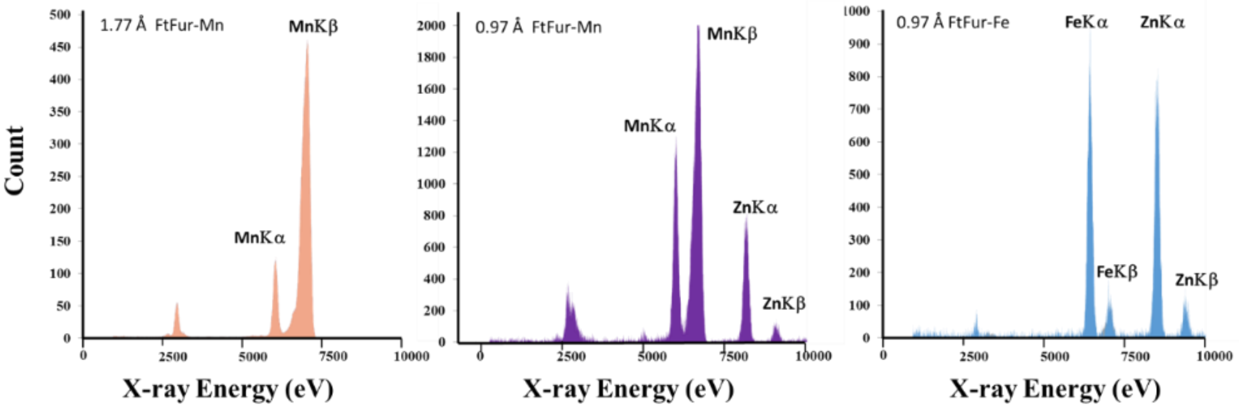

B

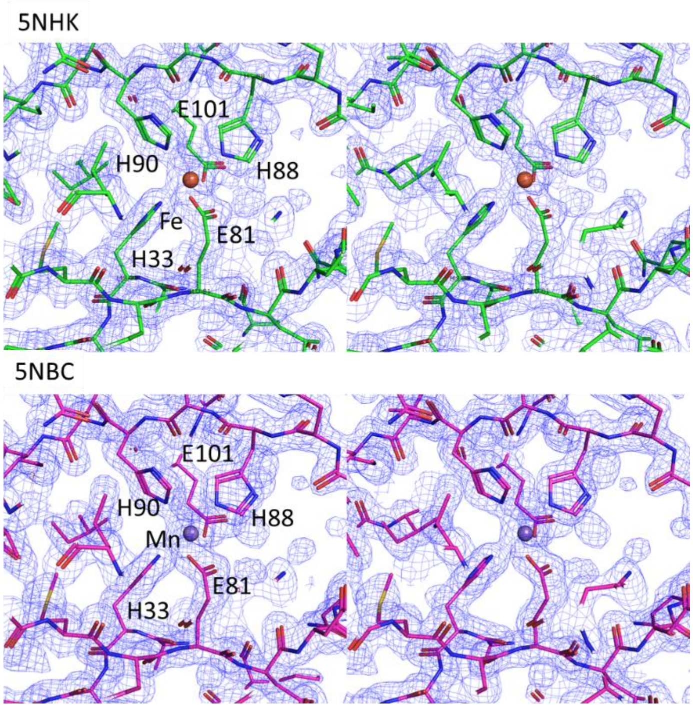

Supplementary Figure 3. Crystallographic data of FtFur structures and fluorescence spectra.

Panel A. Fluorescence spectra recorded at 1.77 Å and 0.97 Å to confirm the presence of Fe, Zn and Mn atom in protein crystals before X-ray diffraction.
Panel B. Stereo views of the Fe and Mn metal binding sites of the 5NHK and 5NBC structures, respectively. Stereo mode prepared with the Will Eye Stereo Program inside Pymol 2.1.0 (Sigma 1.5; Carve 2.0; mesh 0.1; Stereo shift -6; Stereo angle 2.1)

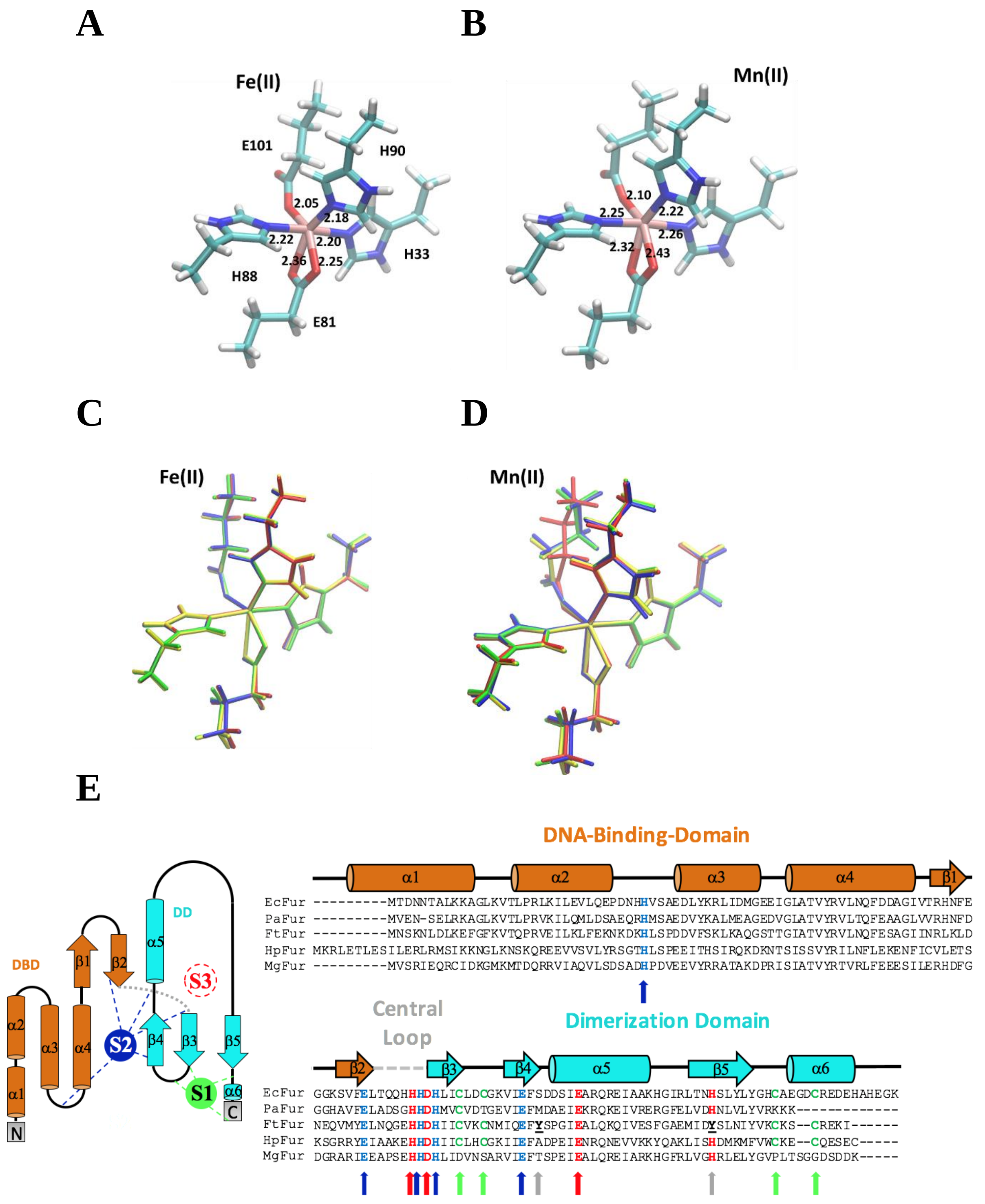

Supplementary Figure 4. Structures of metal binding sites and sequence alignments.
Panels A and B. FtFur Metal binding sites – DFT B3LYP Optimization under High spin for S2 sites filled with Fe(II) and Mn(II) respectively.
Panels C and D. Superimposition of DFT optimized structures of the metal sites in the 4 subunits, for S2 sites filled with Fe(II) and Mn(II) respectively.
Panel E. Sequence alignment of 5 Fur proteins of known structures and location of amino acids implicated in the S1 (Green), S2 (Blue) and S3 sites (red). Y103 and Y125 from FtFur are identified by grey arrows. Ec=*Escherichia coli* (DBD Xray structures only [5]; Pa: *Pseudomonas aeruginosa* [6]; Ft: *Francisella tularensis* (this work); Hp: *Helicobacter pylori* [7] and Mg: *Magnetospirillum gryphiswaldense* [8].

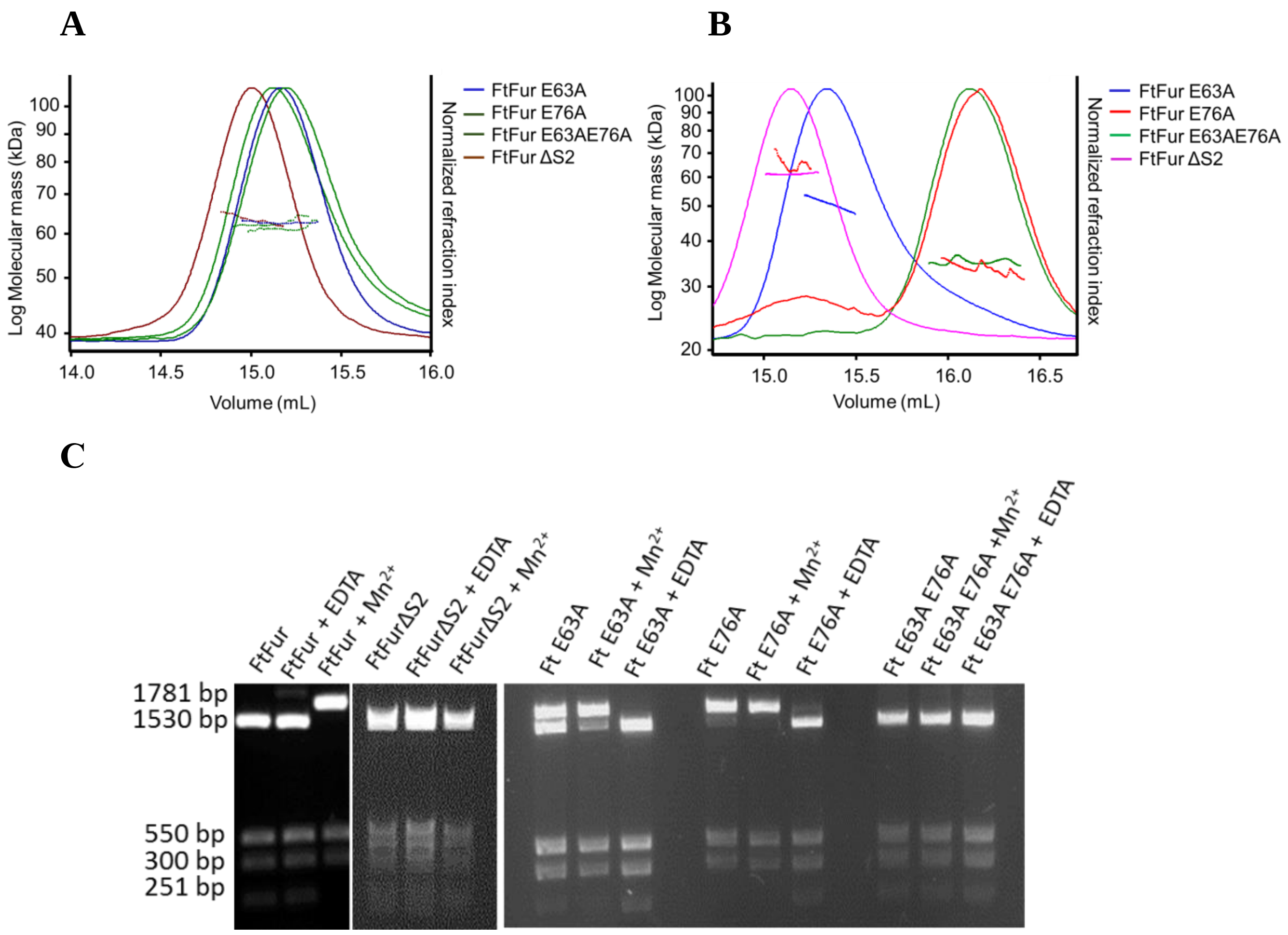

Supplementary Figure 5. Study of tetramer dissociation by SEC-MALLS-RI analysis on FtFur E63A, E76A and E63AE76A mutants and activity assays.

Panel A. SEC-MALLS analysis experiments are done in the presence of 150 mM NaCl, all constructs present the same MW (around 64 kDa) corresponding to a tetramer in solution.

Panel B. SEC-MALLS analysis experiments are done in the presence of 500 mM NaCl. FtFur-WT presents a MW of 64 kDa corresponding to a tetramer in solution. E63A mutant shows a non-symmetric peak at 48 kDa MW corresponding to a mixture of tetramer and dimer. E76A present two peaks. The major peak corresponds to a MW of 32 kDa for a dimer in solution, the minor peak presents a MW of 64kDa corresponding to a tetramer. Finally, the double mutant E63AE76A presents one single symmetric peak at 32 kDa corresponding to a stable dimer in solution. This experiment has been repeated three times and validates the major role of these two amino acids in the stabilization of the tetramer.

Panel C. Nuclease assay on FtFur, FtFurΔS2, FtFurE63A, FtFurE76A, FtFurE63AE76A performed as previously described[4]. The FtFurΔS2 and the FtFurE63AE76A mutants were not able to bind DNA contrarily to the WT, FtFurE63A and FtFurE76A mutants, in the conditions of the assay.

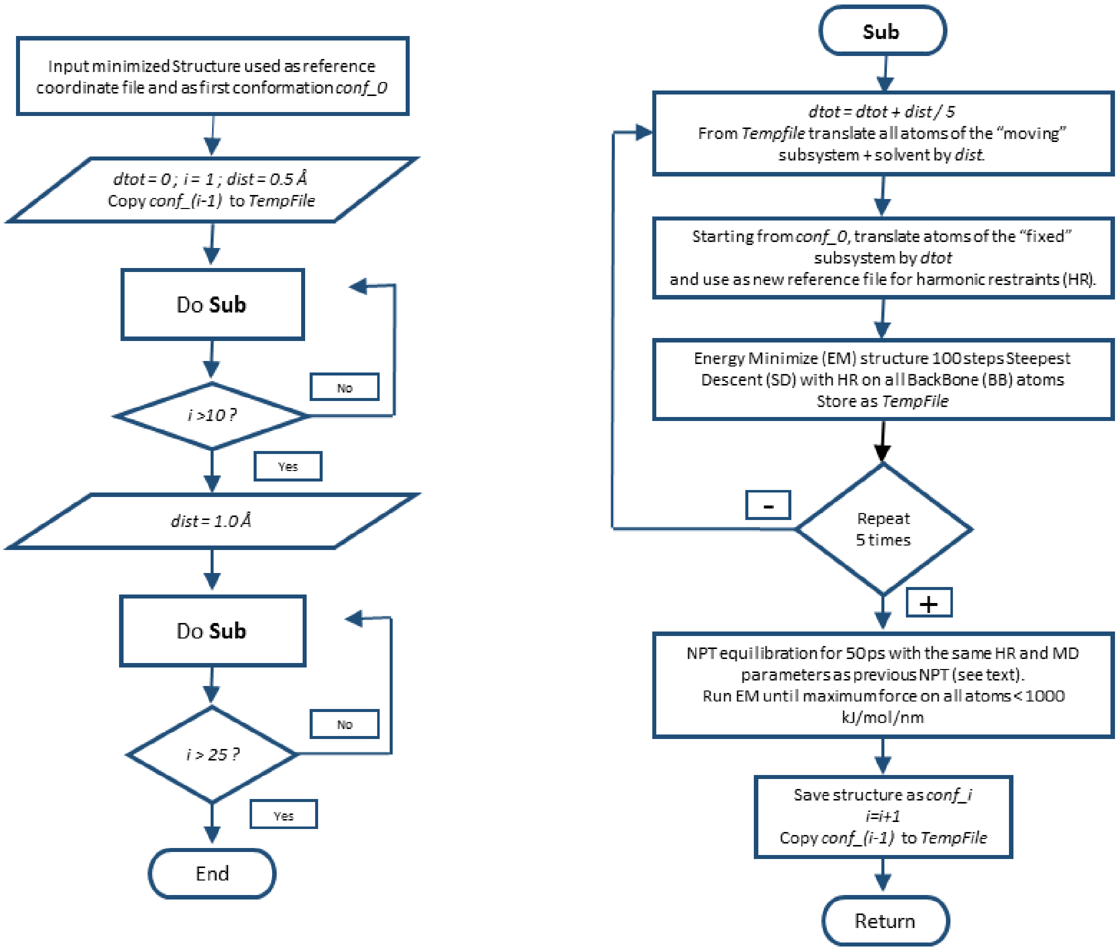

Supplementary Figure 6. Algorithm for the preparation of initial coordinates for the computation of free energy profiles.
The algorithm consists of a main subprogram doing the translations and pre-equilibrations, which is called twice: 1) 10 first windows corresponding to 0.5 Å translations followed by 2) 15 windows of 1 Å translations. Three variables are defined "dtot", the total translation distance for the "moving" subsystem, "i", the conformation counter and "dist", the current translation distance in Å.

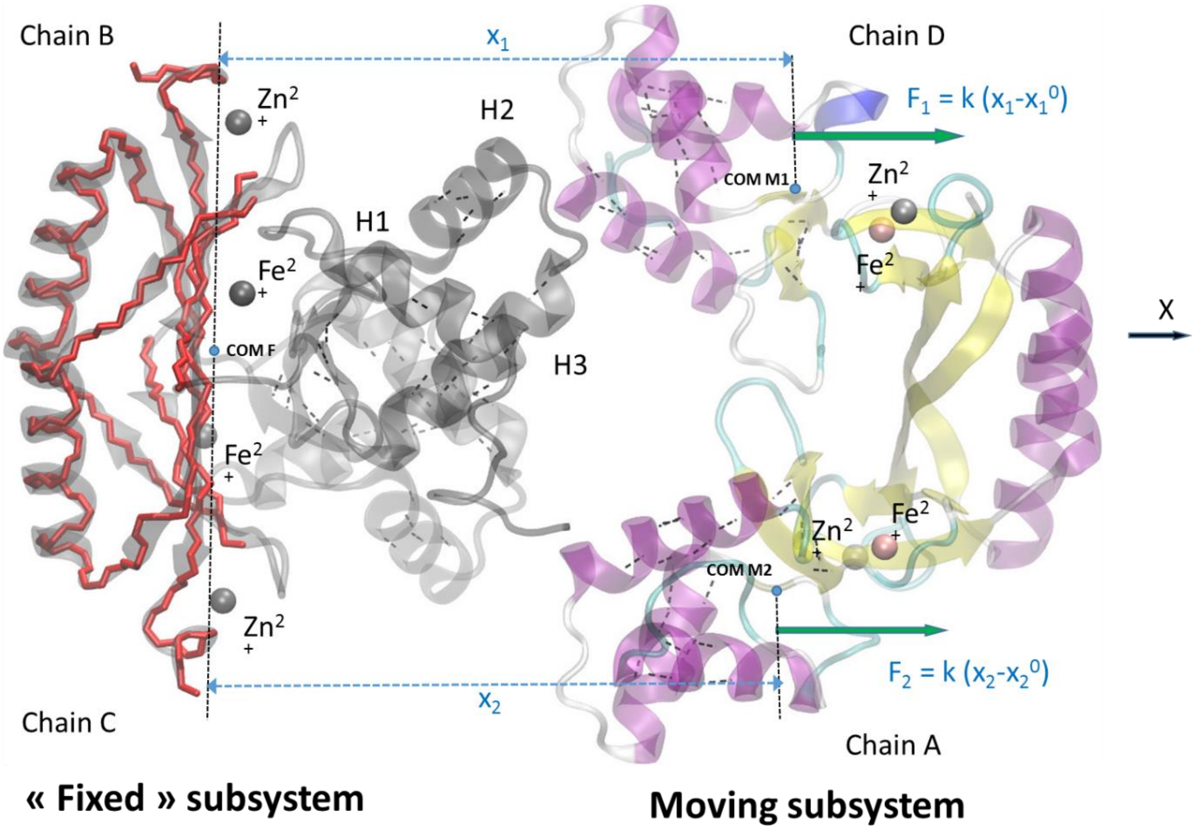

Supplementary Figure 7. Structure of the FtFur tetramer corresponding to the last conformation in the pulling process.

The moving subsystem (Chains A and D) has been translated (10*0.5+15*1.0) =20 Å from the initial tetramer along the X direction. Centers of mass of the "fixed" subsystem and the two subunits of the moving Ft dimer are indicated. The moving subsystem is constituted of two Ft subunits: 1 and 2. Forces are applied on their centers of mass to pull them away or maintain them at a fixed distance of the "fixed" subsystem. Part of the backbone of the "fixed" system highlighted in red is subject to position restraints during the pulling process. Black dashed lines show hydrogen bonds in the secondary structures which are maintained using NOE type distance restraints. 8 metal cations are shown as spheres. Water and counter ions surrounding the system (in a 105.3*80.3*65.4 Å^3 periodic box) are not shown for clarity.

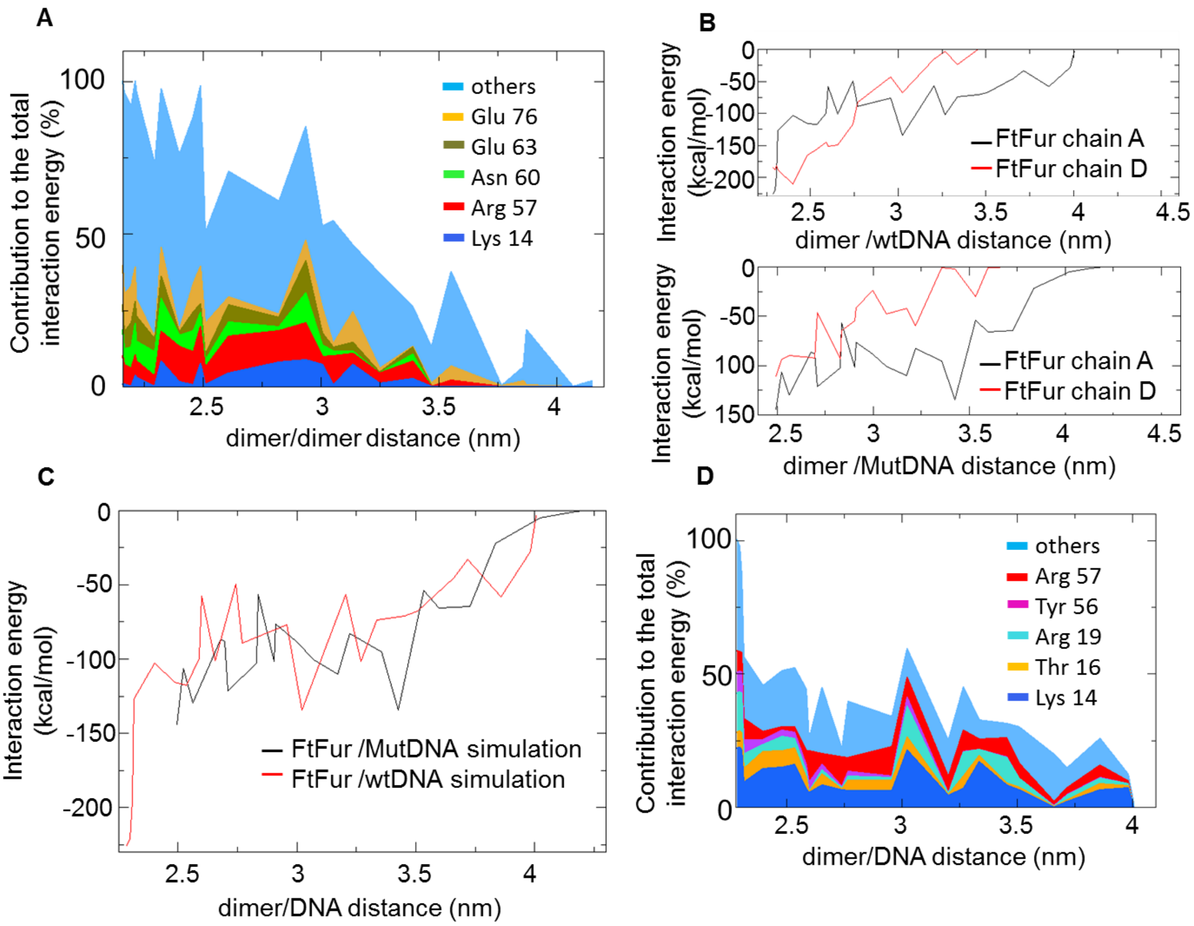

Supplementary Figure 8. Important residues in the interactions and their comparative energy contribution.

Panel A. The five major contributors to the interaction energy between chain A and the "fixed" dimer in the FtFur tetramer simulation. Same legend as in Fig. 2. On average, 5 residues contribute to around 30% of the total interaction energy (with a maximum of 56% at 2.93 nm). The maximum total interaction energy for all residues over all 26 windows is calculated first, then the contribution of each residue is normalized with respect to this maximum. The plot shows the corresponding percentage of the maximum interaction energy for selected residues. The blue surface corresponds to the sum of the contributions of all residues others than those selected.

Panel B. Up: Comparison of average interaction energies between Fur chains A and D and wtDNA during the translation of Fur away from DNA. Chain D loses its interaction with DNA faster than chain A. Down: Comparison of average interaction energies between Fur chains A and D and mutDNA during the translation of Fur away from DNA. Chain D loses its interaction with DNA very fast. (Averages were calculated over 15000 points).

Panel C. Comparison of average interaction energies between Fur chain A and DNA in the Fur/wtDNA and Fur/mutDNA simulations. Interaction energies between FUR chain A and wt or mut DNA are similar.

Panel D. The five major contributors to the average interaction energy between Fur chain A and DNA in the FtFur/wtDNA simulation. Same legend as in Fig. 2. On average, 5 residues contribute to 59% of the total interaction energy (with a maximum of 86% at 2.74 nm).

Supplementary Tables

Supplementary Table 1. Data collection, phasing and refinement statistics for the structure of Mn-FtFur.

	Mn-FtFur (5NBC)	
**Data collection**		
Space group	P21	P21
Cell dimensions		
a, *b*, *c* (Å)	53.15, 89.93, 63.84	53.15, 90.0, 63.95
α, β, γ (°)	90.00, 93.59, 90.00	90.00, 93.48, 90.00
	Peak	*Remote*
Wavelength (Å)	1.7761	0.9797
Resolution (Å) [a]	50.0 - 2.1 (2.14 - 2.1)	50.0 - 1.7 (1.74 - 1.7)
R_{meas} [a, b]	17.6 (135.0)	8.9 (99.2)
$I/\sigma(I)$ [a]	10.2 (1.60)	11.6 (1.31)
$CC_{1/2}$ [a]	99.5 (57.8)	99.6 (64.4)
Completeness (%) [a]	99.3 (92.3)	99.7 (48.8)
Redundancy [a]	7.2 (6.4)	3.5 (2.4)
**Refinement**		
Resolution (Å)	*N.A.*[c]	42.1 - 1.7
No. reflections		64585
R_{work} / R_{free}		21.7/24.4
No. atoms		
Protein		4138
Ligand/ion		8 (4 Zn atoms and 4 Mn atoms)
Water		402
B factors		
Protein (Å^2)		24.1
Ligand/ion (Å^2)		20.9
Water (Å^2)		31.3
R.m.s deviations (Å^2)		
Bond lengths (Å)		0.007
Bond angles (°)		0.88

[a]Values in parentheses are for highest-resolution shell.

[b]R_{meas}, redundancy-independent merging R-factor

[c] Structure of Mn-FtFur has been solved by the MAD method using 1 crystal, and refined with the remote diffraction data only

Supplementary Table 2. Data collection and refinement statistics for the structure of Fe-FtFur.

	Fe-FtFur (PDB code 5NHK)
**Data collection**	
Space group	P21
Cell dimensions	
a, *b*, *c* (Å)	53.35, 90.04, 64.05
α, β, γ (°)	90.00, 93.53, 90.00
Resolution (Å) [a]	50.0 - 1.8 (1.9 - 1.8)
R_{meas} [a, b]	9.6 (75.2)
$I/\sigma(I)$ [a]	10.8 (2.1)
$CC_{1/2}$ [a]	99.6 (64.4)
Completeness (%) [a]	99.2 (99.6)
Redundancy [a]	3.0 (3.0)
**Refinement**	
Resolution (Å)	42.2 - 1.8
No. reflections	55477
R_{work} / R_{free}	20.4/24.3
No. atoms	
Protein	4237
Ligand/ion	8 (4 Zn atoms and 4 Fe atoms)
Water	507
B factors	
Protein (Å^2)	22.2
Ligand/ion (Å^2)	20.0
Water (Å^2)	29.4
R.m.s. deviations	
Bond lengths (Å)	0.008
Bond angles (°)	0.89

[a]Values in parentheses are for highest-resolution shell.
[b]R_{meas}, redundancy-independent merging R-factor

Supplementary Table 3. Comparison of metal-ligand distances between FtFur-Mn and FtFur-Fe. Distances are calculated from the X-ray structures with Coot 0.8.2

Mn-FtFur / Fe- FtFur

	Site 1			
	Zn in dimer 1		Zn in dimer 2	
	Chain A	Chain D	Chain B	Chain C
C93	2.30/2.31	2.36/2.37	2.33/2.34	2.35/2.35
C96	2.31/2.32	2.30/2.32	2.31/2.26	2.32/2.31
C133	2.33/2.30	2.32/2.30	2.36/2.31	2.28/2.29
C136	2.32/2.30	2.37/2.30	2.31/2.31	2.30/2.30
	Site 2			
	Mn/Fe in dimer 1		Mn/Fe in dimer 2	
	Chain A	Chain D	Chain B	Chain C
H33 (Nε2)	2.31/2.26	2.26/2.26	2.29/2.27	2.28/2.26
E81(Oε1)	2.32/2.19	2.30/2.20	2.27/2.10	2.35/2.21
(Oε2)	2.32/2.31	2.34/2.34	2.30/2.25	2.29/2.31
H88 (Nε2)	2.31/2.29	2.36/2.26	2.31/2.30	2.32/2.27
H90 (Nε2)	2.23/2.22	2.26/2.21	2.30/2.26	2.31/2.24
E101 (Oε2)	2.35/2.03	2.20/2.00	2.32/2.11	2.16/1.97

Supplementary Table 4. Strains, plasmids, and primers used in this study.

Strain, plasmid, or primer	Genotype, description, or sequence	Source or reference
**Strains**		
CHUGA-Ft6	*F. tularensis subsp. holarctica*, human strain from blood sample (Verdun hospital)	This study
CHUGA-Ft6Δfur	CHUGAFt66 with in-frame deletion of FTA_1939 (fur)	This study
CHUGA-Ft6Δfur/pfur	CHUGAFt66Δfur trans complemented with fur gene and promotor region on plasmid pMP 828	This study
**Plasmids**		
Pmp812furpart	suicidal plasmid pMP 812 with 1000 pb regions upstream and downstream *fur g*ene	Lovullo *
Pmp 828fur+	shuttle plasmid containing *fur* gene of *F. tularensis subsp. holarctica* and its promotor region	Lovullo *
**Primers**		
**LeftfurF**	**GATCGGATCCTCTGCAGTTGTATAATGCCG**	This study
**LeftfurR**	**CTATTAGATAGTTTTATAAGTTC**	This study
**RightfurL**	**TATGCTTTTCGAATAATCTTTATATCAGAACTTATAAAACTATCTAATAGAC GCAATAATCACTATCCAG**	This study
**RightfurR**	**TAGCGATATCGTGATACCAAATTTTATATTGCTC**	This study
**CompfurL**	**GCTCATAAACACTTGGATCCTAATCAAGAAGA**	This study
**CompfurR**	**AAGCTTAAATTTAGAGATATCAGAAAAGCTGTT**	This study

CHUGA-Ft6 was isolated at Verdun Hospital (France) from a blood sample of a patient with typhoidal tularemia [1]. * Lovullo et al [2,3].

Supplementary Table 5. Oligonucleotide sequences used in Gene expression by qRT-PCR experiment.

Name	Sequence
fur: forward	GGAATCGAAGCTCTGCAAAA
fur: reverse	TTCACGACAGGATTTGCATT
figA: forward	AACTGCTCCCCATTGCTCTA
figA: reverse	TTGGCAATGGTTAACTGCAA
16S-RNA: forward	TTTCACCTTTGAGCTGTTGC
16S-RNA: reverse	CCTTTGGCAAATTCAATAGAAAC

Supplementary Methods

Molecular Modelling of the FtFur tetramer

The pdb file corresponding to the Xray structure of the FtFur tetramer was prepared for gromacs (GROningen MAchine for Chemical Simulations) version 5.1.2 [9] with pdb2gmx using gromos54a7 united atom force field [10]. All histidine residues were given type HISA with protonated Nδ1 atom. In all 4 protein subunits cysteines 93, 96, 133 and 136 were deprotonated and given a total charge of -0.75 (-0.05 for Cα, -0.15 for Cβ, -0.55 for S). This charge can be compared to that of the CYS residue in gromos54a7 force field, with total charge of -0.5. A value of -0.75 was adopted instead in this work, high enough to stabilize a tetracoordinated Zinc ion, providing correct orientation of the cysteines and proper metal environment during further simulations. (Use of a -0.5 charge led to the escape of the metal from its binding site).

Construction of the MgFur dimer DNA complex

The structure of *Magnetospirillum gryphiswaldense* (4RB1) was used as initial model of a Fur per DNA complex. The Fur dimer includes residues 20 to 133 and the DNA double strand is composed of CGGATAATGATAATCATTATCGC. First, an initial model (Mod1) of the MgFur dimer per DNA complex with 4 iron ions and 2 bound water molecules (included in structure 4RB1) was built and slightly energy minimized with CHARMM [11] using the all-atom "par_all27_prot_na" parameter file for proteins and nucleic acids. Second, another initial model (Mod2) of the protein dimer alone was built starting from the same PDB but with the extended atom "param19" force field for proteins. Then, an initial structure of the protein DNA complex compatible with the Gromacs force field was created from protein dimer from Mod2 and DNA from Mod1. The structure was oriented so that DNA was centered at the origin with its principal axis along Y. We noticed that the MgFur dimer in this structure sits asymmetrically on the DNA fragment with K15 in chain A interacting with CYT21 and K15 in chain B extending over CYT1. It was consequently decided to move the last two GC bases upstream leading to a GCCGGATAATGATAATCATTATC fragment. The new position of the 2 moved nucleotides was controlled with VMD and regularized through 1000 steps of energy minimization with CHARMM. This new DNA fragment offers the advantage of providing good interactions for all residues in the Fur dimer while still including the full length consensus Fur box GATAATGATAATCATTATC.

Construction of the FtFur dimer per wild type DNA complex

Structure of FtFur in the presence of iron (this work) from ASP7 to ARG137 was used for the Fur/wtDNA simulation. In the FtFur+DNA structure, the model built from MgFur was used as template to align FtFur. FtFur was superimposed on MgFur already sitting on the Fur box using least square fit of matching atom positions. Backbone atoms from corresponding structured parts (helices and sheets) of the two protein were selected for this structure superposition for a final rms difference of 4 Å.

Construction of the FtFur dimer per mutated DNA complex

The mutated DNA (mutDNA) sequence GCCGGATA**C**TGATA**G**TC**C**T**G**ATC contains four mutations with respect to the Fur-box (A9 to C, A15 to G, A18 to C and T20 to G). Initial coordinates for mutDNA were taken from the previously obtained structure of wild type DNA by simple matching of corresponding heavy atoms and building of missing hydrogens with CHARMM, followed by 1000 steps energy minimization. Initial direct superimposition of the Fur dimer onto this mutated DNA showed that relatively large structure modifications could occur in the region of the mutations. Consequently, a 100 ps NPT simulation of solvated

mutDNA was run to release the strains in the structure. This last structure was superimposed on the initial model built in the presence of FtFur to ensure optimal orientation. Finally, initial coordinates for the FtFur dimer per mutated DNA complex (FtFur/mutDNA) were obtained by concatenating coordinates of the protein in the FtFur/wtDNA complex and those of the reoriented mutDNA.

Computational details of the solvation and equilibration

For the tetramer, the maximum dimensions of the protein were calculated, 28 Å were added in the +X direction to allow for its further translation. Then, a simulation box 5 Å larger than these dimensions in all directions was created and the tetramer centered in the simulation box with "editconf". Initial dimensions of the box were 10.53*8.03*6.54 nm. "gmx solvate" was used to add solvent (SPC water) to the protein from a preequilibrated water box (spc216.gro) for a total of 15586 water molecules. 37 sodium and 33 chloride ions were added (replacing 70 water molecules) for a total ionic force of 0.1 mol.L^{-1} and a zero total charge of the 52114 atom system.

For the FtFur/wtDNA complex, initial dimensions of the box were 9.28*8.91*6.35 nm. 15505 water molecules were added of which 77 were replaced by sodium and 32 by chloride ions to ensure neutrality and a total ionic force of 0.1 mol.L^{-1} for a total of 50117 atoms in the system.

For the FtFur/mutDNA complex, initial dimensions of the box were 9.64*9.22*6.39 nm. 16680 water molecules were added of which 79 were replaced by sodium and 34 by chloride ions to ensure neutrality and a total ionic force of 0.1 mol.L^{-1} for a total of 53642 atoms in the system.

All three systems (FtFurTetra, FtFur/wtDNA, FtFur/mutDNA) were energy minimized until the force on all atoms was less than 1000 kJ.mol^{-1}.$Å^{-1}$. Heavy atoms of the protein were restrained to their initial position to prevent big structural changes before the production run with a force constant of 10 kJ.mol^{-1}.$Å^{-2}$. Then we ran the system under NPT conditions at 310 K and 1 atm for 100 ps, with Berendsen temperature and isotropic pressure coupling [12]with τ_T = 0.1 ps, τ_P = 2 ps and compressibility = 4.5×10^{-5} bar^{-1} (same conditions in the NPT simulation used to equilibrate mutDNA).

Computation of Free energy profiles

Free energy profiles for the extraction (by translation along a fixed direction: Ox) of one FtFur dimer from the tetramer (dimer of dimers) and of FtFur from DNA were computed.

The FtFur tetramer is organized around 4 subunits with chain names B and C for the fixed dimer and A and D for the translated dimer. The simulations will thus include a "moving" subsystem (FtFur dimer, chains A and D) and a "fixed" subsystem (FtFur dimer, chains B and C, wtDNA or mutDNA).

The profiles were built using the "umbrella sampling" technique and result from the overlapping of 26 computation windows, one for each translation distance.

The simulation protocol for the equilibration of umbrella sampling windows is best described in supplementary Figure 6.

As a result of this initialization protocol, 26 structures "conf_i" were generated yielding initial positions for the translated dimer prior to the potential of mean force calculation.

Then the umbrella sampling calculation, itself, consisted of 26 repeats of:

- Reading of initial structure "conf_i" and reference structure "conf_0" for harmonic restraints;

- Running 100 ps NPT equilibration with position restraints on the "fixed" subsystem and distance restraints on the protein. The "moving" subsystem was subject to two harmonic biasing forces (umbrella potential) applied between the centers of mass of the 2 Fur

dimer subunits and the center of mass of the “fixed” subsystem with force constants of 500 kJ mol^{-1} nm^{-2}.

- Running 10 to 15 ns NPT production simulations with same restraints and biasing potential. These simulations were concatenated from series of 1 to 2 ns runs allowing us to check the convergence of the PMFs. Harmonic biasing forces were applied along the X direction only (direction of the translation).

Position restraints were applied on backbone atoms of the “fixed” subsystem for residues K28 to S35 (Loop between H1 and H2) and for all residues N83 to E138 (C-Terminal) except V94 to M98 possibly interacting with the “moving” subsystem during the translation. Force constants of 10 kJ $mol^{-1}Å^2$ in all directions were used.
NOE type distance restraints were added to maintain the secondary structure of the protein (both “fixed” and “moving” parts in the case of the tetramer). All backbone hydrogen bonds between N-H and O=C for residues T16 to K24 (H1), S35 to K44 (H2) and G51 to E63 (H3) plus the short beta-sheet (I67—N83, N69—E81, L71—M79) were restrained between 1.8 and 2.0 Å with force constants of 20 kJ $mol^{-1}Å^2$.

In both equilibration and production umbrella sampling MD simulations, the system was simulated under NPT conditions. Temperature was fixed at 310 K with Temperature coupling using a Nose-Hoover extended ensemble with τ_T = 0.5 ps, Pressure was controlled at 1 atm with extended-ensemble Parrinello-Rahman isotropic pressure coupling with τ_P =1 ps and compressibility = 4.5 × 10^{-5} bar^{-1} [13]. Again, a time step of 2 fs was used.

Fitting of the free energy profiles
Data points corresponding to the outputs of wham were fitted with a sum of 1, 2 or 3 sigmoid functions with R [14]:

$$\mathrm{S(x)} = a + \frac{\mathrm{b}}{1 + e^{(-c*(x-d))}}$$

After a first fit with the raw data, the energy offset (a in equation) was subtracted from the final energy profiles.

Supplementary References

1. Maurin, M., Pelloux, I., Brion, J.P., Del Bano, J.N. & Picard, A. Human tularemia in France, 2006-2010. *Clin Infect Dis* **53**, e133-41 (2011).
2. LoVullo, E.D., Molins-Schneekloth, C.R., Schweizer, H.P. & Pavelka, M.S., Jr. Single-copy chromosomal integration systems for Francisella tularensis. *Microbiology* **155**, 1152-63 (2009).
3. LoVullo, E.D., Sherrill, L.A. & Pavelka, M.S., Jr. Improved shuttle vectors for Francisella tularensis genetics. *FEMS Microbiol Lett* **291**, 95-102 (2009).
4. Perard, J. et al. Quaternary Structure of Fur Proteins, a New Subfamily of Tetrameric Proteins. *Biochemistry* **55**, 1503-15 (2016).
5. Pecqueur, L. et al. Structural changes of *Escherichia coli* ferric uptake regulator during metal-dependent dimerization and activation explored by NMR and X-ray crystallography. *J Biol Chem* **281**, 21286-95 (2006).
6. Pohl, E. et al. Architecture of a protein central to iron homeostasis: crystal structure and spectroscopic analysis of the ferric uptake regulator. *Mol Microbiol* **47**, 903-15 (2003).
7. Dian, C. et al. The structure of the *Helicobacter pylori* ferric uptake regulator Fur reveals three functional metal binding sites. *Mol Microbiol* **79**, 1260-75 (2011).
8. Deng, Z. et al. Mechanistic insights into metal ion activation and operator recognition by the ferric uptake regulator. *Nat Commun* **6**, 7642 (2015).
9. Abraham, M.J. et al. GROMACS: High performance molecular simulations through multi-level parallelism from laptops to supercomputers. *SoftwareX 1* **11**, 19-25 (2015).
10. Schmid, N. et al. Definition and testing of the GROMOS force-field versions 54A7 and 54B7. *Eur Biophys J* **40**, 843-56 (2011).
11. Brooks, B.R. et al. CHARMM: a program for macromolecular energy, minimization, and dynamics calculations. *J. Comp. Chem.* **4**, 187-217 (1983).
12. Berendsen, H.J.C., Postma, J.P.M., DiNola, A. & Haak, J.R. Molecular dynamics with coupling to an external bath. *J. Chem. Phys.* **81**, 3684-3690 (1984).
13. Parrinello, M. & Rahman, A. Polymorphic transitions in single crystals: A new molecular dynamics method. *J. Appl. Phys* **52**, 7182-7190 (1981).
14. R-Development-Core-Team. R: A language and environment for statistical computing. (ed. Computing, R.F.f.S.) (Vienna, Austria, 2008).
